# Continuous Flow Depolymerization of Polycarbonates and Poly(lactic acid) Promoted by Supported Organocatalysts

**DOI:** 10.1002/cssc.202500420

**Published:** 2025-04-25

**Authors:** Madeleine Edge, Neha Yadav, Ali Al Rida Hmayed, Andrew P. Dove, Arianna Brandolese

**Affiliations:** ^1^ School of Chemistry University of Birmingham Edgbaston B15 2TT UK

**Keywords:** continuous flow, depolymerization, polymer chemical recycling, polymers, supported organocatalysts

## Abstract

Mechanical recycling methods are a simple and effective approach to recycling plastics, but they often result in a direct reduction in the quality of the virgin polymer. Alternatively, chemical recycling of plastic waste provides a closed‐loop pathway that could offer a solution to the current end‐of‐life mismanagement of plastics. However, harsh reaction conditions, scalability, and product purification can limit the applicability of this process on a large scale. Here, an organocatalyzed continuous flow depolymerization strategy is proposed for two soluble, commonly used plastics, poly(lactic acid) (PLA) and bisphenol A polycarbonate (BPA‐PC). This process used glycolysis to upcycle PLA to alkyl lactate and BPA‐PC to bisphenol A and ethylene carbonate under mild reaction conditions (up to 60 °C). The complete depolymerization of both polymers is initially performed under batch conditions, allowing the solvents and catalysts to be screened. The process is further extended under continuous flow to explore catalyst stability and process scalability. Finally, it is demonstrated that alkyl lactate, bisphenol A and ethylene carbonate can be produced from waste polycarbonate and PLA, thus providing safe and economical access to these species through continuous flow depolymerization of plastic waste.

## Graphical abstract

The chemical recycling of plastic waste plays a crucial role in promoting a circular economy. This work reports on the use of recyclable heterogeneous organocatalysts to promote common polycarbonate and polyester glycolysis. Poly(lactic acid) and bisphenol A polycarbonate are here depolymerized at low temperatures in a continuous approach, proving beneficial for longer catalyst stability and easy scale‐up of the process.

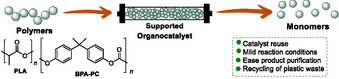

## 1 Introduction

Plastics have become an indispensable part of our daily lives. Every year more than 400 million tons of plastics are produced, yet only 9% is currently effectively recycled; most is incinerated or ends up in landfills.^[^ ^1^ ^]^ Additionally, the majority of our commodity materials are used in a linear lifecycle as they have been continually developed with performance and cost as the key drivers of innovation. A linear lifecycle represents a loss of inherited material value, therefore, solutions that can reduce waste generation and circularize the material value of plastics are highly sought after.^[^ ^2^, ^3^ ^]^ Post‐consumer plastic waste (PCPW) represents a material value that can be enhanced through recycling. At present, plastic recycling is primarily focused on mechanical recycling methods and more recently through advanced recycling such as pyrolysis or thermal recycling.^[^ ^4^, ^5^ ^]^ Mechanical recycling is widely implemented, easy to access, and cheap to run; however, the quality in terms of mechanical and thermal properties of the recycled material is affected, leading overall to a downcycling process.^[^ ^6^, ^7^ ^]^ Hence, chemical recycling to monomers (CRM) offers an alternative strategy to promote plastic recycling.^[^ ^8^, ^9^, ^10^, ^11^, ^12^, ^13^, ^14^ ^]^ Chemical recycling of condensation plastics has been recently extensively explored through alcoholysis, where the alcohol acts as a nucleophilic reagent.^[^ ^15^, ^16^, ^17^, ^18^, ^19^, ^20^, ^21^ ^]^ This process enables the isolation of pure monomers and oligomers from waste plastics, and their subsequent re‐polymerization yields materials of virgin‐grade quality. Furthermore, CRM offers the potential for infinite virgin‐grade recycling and ease contaminant removal,^[^ ^22^ ^]^ *en route* to a circular economy.^[^ ^8^ ^]^

Developing efficient, selective, and low‐energy chemical recycling processes represents a timely challenge to achieve a sustainable plastics economy. Desirable depolymerization conditions include mild temperatures, short reaction times, and green and recyclable catalysts.^[^ ^23^, ^24^ ^]^ Thermally stable organocatalysts have been explored for the CRM of PCWP, including poly(ethylene terephthalate) (PET), poly(lactic acid) (PLA), and bisphenol A polycarbonate (BPA‐PC).^[^ ^25^, ^26^, ^27^ ^]^ Protic ionic salts, for example, 1,5,7‐triazabicyclo[4.4.0]dec‐5‐ene: methanesulfonic acid (TBD: MSA) have been employed as a consequence of their higher stability and activity in promoting polymer chemical recycling.^[^ ^17^, ^28^ ^]^

When used as a sole catalytic system, organocatalysts present limitations, such as low thermal stability and air sensitivity.^[^ ^29^ ^]^ Using a homogeneous catalyst produces issues with recoverability and, therefore, catalytic recycling. Supporting the catalyst on an insoluble polymer *via* covalent or non‐covalent interactions therefore creates a heterogeneous system that can be recovered *via* filtration or centrifugal force.^[^ ^30^ ^]^ This inert material support improves catalyst recycling and stability, with immobilized catalysts also being compatible with a continuous flow approach.^[^ ^31^ ^]^ The use of a silica‐supported TBD organocatalysts catalyst has been recently reported by Kupai and co‐workers to promote BPA‐PC depolymerization, achieving excellent bisphenol A (BPA) isolated yield (94%).^[^ ^32^ ^]^

To be industrially relevant, the CRM of a commercial polymer like PLA or BPA‐PC needs to be scalable, the depolymerized monomer must be easily recoverable, and the process needs to be energy efficient. To this end, a continuous flow approach offers features that allow to perform of a beneficial depolymerization process.^[^ ^33^, ^34^ ^]^ Additionally, flow reactors are easy to scale, and when a packed bed reactor is used, catalysts are easily recycled, further simplifying the product purification process. Despite these advantages, to date, the use of continuous flow for depolymerization reactions has mainly been focused on the depolymerization of lignin and oligosaccharides, with only one report on the CRM of plastic.^[^ ^35^, ^36^, ^37^ ^]^ Junker and co‐workers have recently reported on the use of a continuous flow approach to promote the CRM of low molecular weight PLA (with a number average molecular weight, *M*_*n*_ = 15.8 kDa) to lactide in the presence of commercially available Sn(II) catalyst in tetrahydrofuran (THF) at temperatures of 150–170 °C.^[^ ^38^ ^]^

**Figure 1:**
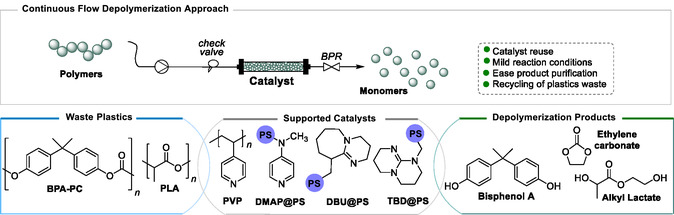
Continuous flow depolymerization of PLA and BPA‐PC with supported catalysts performed in this work. BPR, back pressure regulator.

Herein, we explored the possibility of chemically recycling waste plastics using a supported benign catalyst that can be easily reused and separated from the reaction mixture either through filtration or by being used for the preparation of packed bed reactors (**Figure** **1**). To simplify product recovery, a series of lower boiling point solvents were here explored. Supported organocatalysts (Lewis Bases organocatalysts) and supported biocatalysts (Lipase B from *Candida antarctica*) were first tested under batch conditions, followed by continuous flow experiments. The comparative performance of the heterogeneous catalysts demonstrates that both 1,8‐diazabicyclo[5.4.0]undec‐7‐ene, DBU‐ and 4‐(dimethylamino)pyridine polymer‐bound, DMAP‐based catalysts are productive systems with overall high substrate conversion and selectivity in the glycolysis products both for commercially available BPA‐PC (weight average molecular weight, *M*_w_ = 141 kDa) and PLA (*M*_w_ = 42.1 kDa) and real plastic waste.

## 2 Results and Discussion

### 2.1 Depolymerization under Batch Conditions

A range of solvents able to fully solubilize both BPA‐PC and PLA were explored to perform their depolymerization in the presence of a supported catalyst. At first, the preparation of slurries was considered, however, slurries require diluted solutions that limit the amount of polymer treatable per hour, and the use of peristaltic pumps.^[^ ^7^ ^]^ To overcome these limitations, solvents in which both polymers are completely soluble were investigated (Table S1, Supporting Information). With the aim of recycling the catalyst, commercially available supported organocatalysts and biocatalysts were considered. The catalyst and reaction conditions screening under batch were conducted using ethylene glycol (EG) as the benchmark nucleophile (**Table** **1** and **2**). Based on previous works performed using organocatalyst systems under homogeneous conditions,^[^ ^19^, ^20^ ^]^ we chose several supported organocatalysts derived from *N*‐heterocyclic bases including 1,5,7‐triazabicyclo[4.4.0]dec‐5‐ene supported onto polystyrene (TBD@PS), 1,8‐diazabicyclo[5.4.0]undec‐7‐ene polymer bounded (DBU@PS), 4‐(dimethylamino)pyridine polymer‐bound (DMAP@PS) and poly(4‐vinylpyridine) (PVP) (Figure 1). BPA‐PC and PLA depolymerization were achieved by glycolysis using excess EG, at mild temperatures and the reaction outcome was monitored by ^1^H NMR spectroscopy. PLA conversion to 2‐hydroxyethyl lactate (2‐HEtLa) was determined from the methyl doublet peak at *δ* = 1.45 ppm, and BPA‐PC conversion to bisphenol A (BPA) was calculated from the average integration of the two doublet peaks, which arise at *δ* = 7.10 and 6.74 ppm, respectively.

**Table 1:** Reaction conditions screening for BPA‐PC depolymerization using supported catalysts under batch.a)

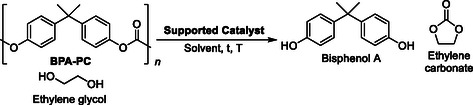				
Entry	Catalyst	Solvent	T [°C]	BPA [%]b)
1	TBD@PS	CH_2_Cl_2_	20	0
2	DBU@PS	CH_2_Cl_2_	20	0
3	TBD@PS	CHCl_3_	60	20
4	DBU@PS	CHCl_3_	60	7
5	TBD@PS	THF	60	32
6	DBU@PS	THF	60	7
7	TBD@PS	DMF	80	71
8	DBU@PS	DMF	80	97
9	TBD@PS	Cyrene	100	n.d.
10	DBU@PS	Cyrene	100	n.d.
11	DMAP@PS	DMF	80	>99
12	DMAP@PS	THF	60	17
13	PVP	DMF	80	50
14	CALB	DMF	80	41

a) 0.25 mmol of BPA‐PC, catalyst (20 mol%), solvent (0.12 M), 24 h;

b) BPA NMR yield determined by ^1^H NMR spectroscopy. n.d., not detected, formation of by‐products.

**Table 2:** Reaction condition screening for PLA depolymerization with supported catalysts under batch.a)

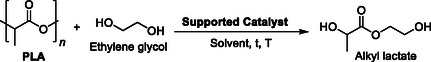				
Entry	Catalyst	Solvent	T [°C]	Alkyl lactate [%]b)
1	TBD@PS	CH_2_Cl_2_	20	7
2	DBU@PS	CH_2_Cl_2_	20	7
3	TBD@PS	CHCl_3_	60	28
4	DBU@PS	CHCl_3_	60	18
5	TBD@PS	THF	60	81
6	DBU@PS	THF	60	>99
7	TBD@PS	DMF	80	93
8	DBU@PS	DMF	80	99
9	TBD@PS	Cyrene	100	n.d.
10	DBU@PS	Cyrene	100	n.d.
11	DMAP@PS	DMF	80	>99
12	DMAP@PS	THF	60	57
13	PVP	THF	60	10
14	CALB	THF	60	<5

a) 1.2 mmol of PLA, catalyst (20 mol%), solvent (0.12 M); 24 h;

b) Alkyl lactate NMR yield determined by ^1^H NMR spectroscopy. n.d., not detected, formation of by‐products.

According to previously reported work, homogenous Lewis Bases organocatalysts proved to be active in both PLA and BPA‐PC depolymerization.^[^ ^25^ ^]^ The choice of a supported catalyst was driven by the possibility of easily recycling and reactivating the catalytic system (e.g., bases can be washed with KOH solution to promote base regeneration)^[^ ^32^ ^]^ and their use in a continuous flow approach. Furthermore, in the present work, we also aimed to develop a scalable depolymerization protocol that occurs under milder reaction conditions (*i.e.*, low temperature), thus being most beneficial for industrial conversion. Therefore, the depolymerization of BPA‐PC (Table 1) was first evaluated at room temperature using CH_2_Cl_2_ as the solvent due to the low boiling point and facile dissolution of both polymers (entries 1 and 2). Under these conditions, however, ^1^H NMR spectroscopy revealed that no depolymerization of BPA‐PC to BPA occurred. Consequently, reactions in CH_2_Cl_2_ at room temperature were discontinued, and chloroform was tested instead, demonstrating promising activity at 60 °C (entries 3 and 4). However, we observed solvent evaporation throughout the reaction as a consequence of chloroform's boiling point (61 °C) being close to the reaction temperature. Hence, we later moved to test more benign solvents, such as THF at 60 °C. Under these conditions, both TBD@PS and DBU@PS led to low conversion (32 and 7%, entries 5 and 6, respectively), suggesting that a higher temperature is required for the depolymerization of BPA‐PC, in line with the literature reports.^[^ ^25^ ^]^

Therefore, the process was explored in dimethylformamide (DMF) at 80 °C, providing a 97% BPA yield (entries 7 and 8). The possibility of opting for a green solvent was also taken into account, testing 2‐methyltetrahydrofuran (Me‐THF), dimethyl carbonate and the sugar‐derived dihydrolevoglucosenone (Cyrene) as alternative green solvents. Both BPA‐PC and PLA showed good solubility in Cyrene, however, in such solvent, the depolymerization of BPA‐PC did not proceed, as Cyrene degraded at high temperatures (entries 9 and 10).

In order to expand the range of catalysts investigated, alternative supported organocatalysts were explored, including DMAP@PS (entries 11 and 12), which showed the best performance in DMF at 80 °C, leading to the complete BPA‐PC depolymerization, while PVP showed only limited conversion (50%; entry 13). Lipase B from *Candida antarctica* (CALB) immobilized onto poly(methyl methacrylate) crosslinked with a divinylbenzene macroporous support (known as Novozym 435) was also chosen as a promising candidate for enzymatic depolymerization. Although enzymes have proved to be able to promote the depolymerization of both fossil‐based and biobased polycondensation polymers,^[^ ^39^ ^]^ commercial CALB has rarely been employed to this end. Herein, Novozym 435 was used in THF at 60 °C, however, only limited BPA yield was achieved in 24 h (41%, entry 14). It is worth noticing that the catalyst systems here tested showed a selective depolymerization of BPA‐PC to BPA and ethylene carbonate (Figure S11 and S12, Supporting Information).

Similarly, batch depolymerization of PLA was investigated by evaluating various catalysts, temperature conditions, and solvent systems (Table 2). As for BPA‐PC, room temperature depolymerization of PLA in CH_2_Cl_2_ with both TBD@PS and DBU@PS resulted in only a minimal alkyl lactate formation (7%, entries 1 and 2). However, increasing the temperature up to 60 °C (in CHCl_3_), led to the expected improvements (28% with TBD@PS and 18% with DBU@PS, entries 3 and 4, respectively).

Moreover, switching to THF as the solvent proved to be beneficial, leading to the complete PLA depolymerization at 60 °C when DBU@PS is used (81% alkyl lactate detected when TBD@PS is used as the catalyst, entries 5 and 6). As previously observed, increasing the reaction temperature to 80 °C proved to be beneficial in promoting PLA complete depolymerization in DMF (entries 7 and 8). The green solvent, Cyrene, was also used, but as previously observed, the solvent degraded at high temperatures (entries 9 and 10). Finally, alternative supported organobases were tested. DMAP@PS promoted PLA completed glycolysis at 80 °C in DMF (entry 11) and 57% alkyl lactate formation in THF (entry 12), while PVP and CALB showed much less activity (10% and <5%, entries 13 and 14, respectively). As observed in the BPA‐PC depolymerization, the organocatalyzed glycolysis of PLA selectively led to the formation of alkyl lactate, while the lactide monomer was not detected (Figure S13 and S14, Supporting Information).

### 2.2 Catalyst Recycling under Batch

**Figure 2:**
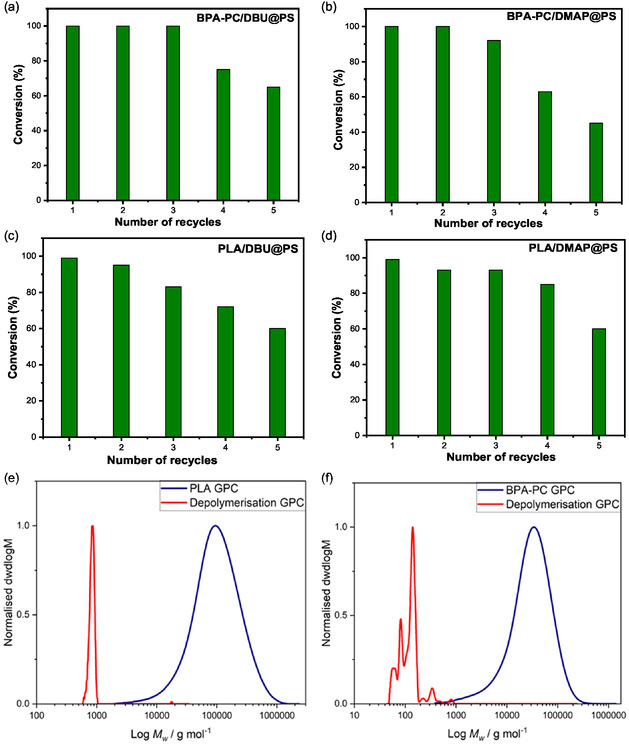
a,b) Recycling experiments under batch conditions for the depolymerization of BPA‐PC with DBU@PS and DMAP@PS in DMF at 80 °C. c,d) Recycling experiments under batch conditions for the depolymerization of PLA with DBU@PS in THF at 60 °C and DMAP@PS in DMF at 80 °C. e) SEC analyses of PLA and PLA depolymerization products. f) SEC analyses of BPA‐PC and BPA‐PC depolymerization products.

Given the relatively mild reaction conditions that were used for depolymerization in batch, we explored the possibility of testing the recyclability of the catalysts employed for both PLA and BPA‐PC depolymerization. Catalyst activity was monitored across five consecutive glycolysis cycles (**Figure** **2**). Since BPA‐PC achieved nearly complete conversion in batch reactions with both DBU@PS and DMAP@PS in DMF at 80 °C, the recycling performance of both catalysts was evaluated. For DBU@PS, >99% conversion was maintained during the first three recycles, but the activity decreased to 70% after five cycles, exhibiting a similar trend for DMAP@PS (Figure 2a,b and Figure S11 and S12, Supporting Information). Since PLA also showed high conversion in comparatively milder reaction conditions with DBU@PS (60 °C), catalyst recycling was similarly investigated in THF (Figure 2c and Figure S13, Supporting Information). As observed in other recycling studies,^[^ ^32^ ^]^ a gradual decrease in catalyst activity occurred after three cycles, indicating the deactivation of DBU@PS. Similarly, the catalytic recycling of DMAP@PS was assessed with PLA in DMF at 80 °C (Figure 2d and Figure S14, Supporting Information). Under these conditions, PLA conversion started to diminish after the first cycle, reaching 60% after five cycles. This gradual reduction in the catalytic activity of both DMAP@PS and DBU@PS could be attributed to the deactivation of the catalysts.

As previously reported, the decline in catalyst activity during BPA‐PC depolymerization has been attributed to catalyst protonation by acidic BPA.^[^ ^32^ ^]^ We also hypothesized that a similar activity decay in PLA depolymerization arose from lactic acid formation, likely caused by trace amounts of water present in both the ethylene glycol and the solvent used. Thus, the possibility of regenerating the catalyst at the end of five reaction cycles by washing them with 6 M KOH was also explored. After careful wash of the supported catalyst to remove traces of KOH, these regenerated catalysts (both DBU@PS and DMAP@PS) were then used to depolymerize both BPA‐PC and PLA in DMF at 80 °C. The ^1^H NMR spectra showed >99% conversion for both BPA‐PC and PLA indicating the successful regeneration of both DBU@PS and DMAP@PS by using such a simple washing process (Figure S15 and S16, Supporting Information).

Finally, depolymerization kinetics were explored and monitored by ^1^H NMR spectroscopy over 24 h, integrating the peak corresponding to the reference proton environment within each product against the ones of the polymers (Figure S17, Supporting Information). The complete depolymerization was also confirmed by size exclusion chromatography (SEC) showing the full depolymerization of these high molecular weight polymers to fractions with a number average molar mass in the order of magnitude of the predicted depolymerization products, although it should be noted that the lowest molecular weight fractions are outside of the calibration window of the instrument used (Figure 2e,f).

### 2.3 Continuous Flow Depolymerization

**Figure 3:**
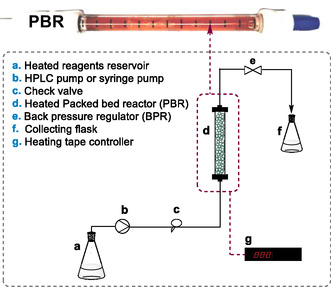
Flow catalysis setup and swollen catalyst gel inside a packed bed reactor (PBR).

The optimized heterogeneous batch glycolysis conditions developed for both BPA‐PC and PLA polymers were chosen as a starting point to explore the continuous flow depolymerization of these polymers. A packed bed reactor (PBR) was thus prepared with both DBU@PS and DMAP@PS. The PBR was surrounded by heating tape to allow it to perform depolymerization at high temperatures, and the reagent reservoir was kept at the same temperature as the reactor system (**Figure** **3**). To optimize the performance of the flow system, several flow catalysis experiments were conducted primarily focusing on the BPA‐PC and PLA conversion (**Table** **3** and **4**, and Table S2, Supporting Information). A solution of BPA‐PC or PLA in THF or DMF, in the presence of ethylene glycol, was let to flow inside this system and the outstream was collected in vials and analyzed through off‐line ^1^H NMR spectroscopy to monitor the polymer conversion.

**Table 3:** Screening of the reaction conditions for BPA‐PC depolymerization under continuous flow conditions using a supported catalyst.a)

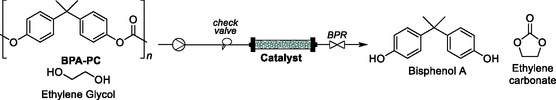					
Entry	Catalyst	T [°C]	Flow rate [mL min^−1^]	*τ* [min]	BPA [%]b)
1	DBU@PS	rt	0.1	40	<5
2	DBU@PS	rt	0.05	80	<5
3c)	DBU@PS	60	0.05	80	35
4c)	DMAP@PS	rt	0.1	40	56
5	DMAP@PS	rt	0.05	80	60
6c)	DMAP@PS	60	0.05	80	>99

a) Reaction conditions: polymer (0.83 mmol, 1.0 equiv), EG (10 equiv), THF (0.06 M), 0.7 g of catalyst (DBU@PS catalyst loading = 1.5–2.5 mmol g^−1^; DMAP@PS catalyst loading = ≈3.0  mmol g^−1^);

b) BPA NMR yield detected by ^1^H NMR spectroscopy, measured when the steady‐state regime was reached;

c) Amount of EG used = 30 equiv. *τ* stands for residence time.

**Table 4:** Screening of the reaction conditions for PLA depolymerization under continuous flow conditions using a supported catalyst.a)

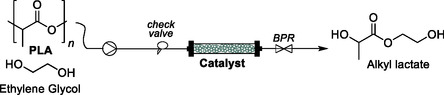						
Entry	Catalyst	Solvent	T [°C]	Flow rate [mL min^−1^]	*τ* [min]	Alkyl lactate [%]b)
1	DBU@PS	THF	60	0.1	40	<5
2	DBU@PS	THF	60	0.05	80	59
3	DBU@PS	DMF	68	0.05	80	62
4	DMAP@PS	THF	60	0.05	80	>99
5	DMAP@PS	DMF	68	0.05	80	>99

a) Reaction conditions: polymer (2.2 mmol, 1.0 equiv), EG (10 equiv), solvent (0.15 M), 0.7 g of catalyst (DBU@PS catalyst loading = 1.5–2.5 mmol g^−1^; DMAP@PS catalyst loading = ≈3.0 mmol g^−1^);

b) Alkyl lactate NMR yield detected by ^1^H NMR spectroscopy, measured when the steady‐state regime was reached. *τ* stands for residence time. Reactions in DMF were conducted at the highest temperature of 68 °C due to operational limitations.

BPA‐PC was tested as the first, and DBU@PS activity was explored in THF at room temperature with 0.1 mL min^−1^ flow rate (Table 3, entry 1. Residence time (*τ*);^[^ ^40^, ^41^ ^]^ *τ* = 40 min). Only minimal polycarbonate conversion was detected over 6 h experiment, so the influence of the flow rate and the temperature was further investigated. Decreasing the flow rate led to an increased residence time, but this did not show any process improvement (entry 2), while the higher temperature (60 °C) proved to be fundamental to promoting the depolymerization (entry 3). DMAP@PS showed better activity at room temperature, leading to 56% of polycarbonate depolymerized to BPA and EC when the steady state is reached (entry 4). Lowering the flow rate led to a slight improvement in the reaction outcome (entry 5), while the increase of the temperature also, in this case, proved to be key to achieving the full glycolysis of BPA‐PC (entry 6). This outcome showed the benefits of the increased mass transfer achieved under continuous flow, leading to higher conversion with respect to the same process conducted under batch in the presence of THF. Furthermore, the use of low‐boiling point solvent aided not only in product purification but also in solvent recycling through distillation. For this reason, the use of THF under continuous flow was favored over DMF.

Similarly, the continuous flow glycolysis of PLA was optimized, exploring the role of the catalyst, solvent, and temperature on the reaction outcome (Table 4). The low solubility of PLA in THF and DMF at room temperature excluded the possibility of performing the reaction at 25 °C. Therefore, DBU@PS was initially tested at 60 °C with 0.1 mL min^−1^ flow rate, showing very little formation of alkyl lactate (entry 1). The effect of the flow rate and, therefore the residence time in this experiment proved to be important in order to achieve a higher alkyl lactate yield (59% recorded when *τ* = 80 min, entry 2). Changing the solvent to DMF did not show any improvements (entry 3), while moving to DMAP@PS led to full alkyl lactate recovery both in THF (at 60 °C, entry 4) and DMF (at 68 °C, entry 5).

**Figure 4:**
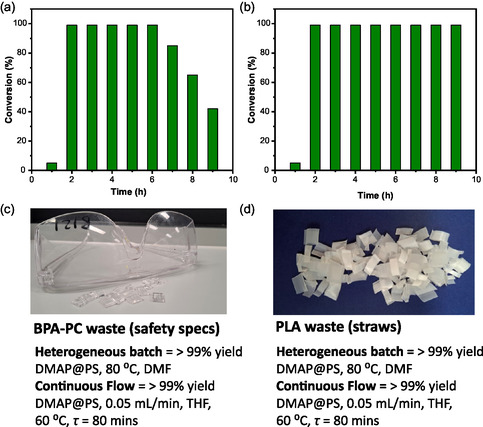
a) BPA‐PC depolymerization under a long‐run continuous flow experiment (reaction conditions Table 3, entry 6); b) PLA depolymerization under a long‐run continuous flow experiment (reaction conditions Table 4, entry 4); c) chemical recycling of BPA‐PC waste; d) chemical recycling of PLA waste.

Once the reaction conditions under CF were optimized (Table 3, entry 6 for BPA‐PC and Table 4, entry 4 for PLA), long‐run experiments were performed to monitor the catalyst stability. Fractions were collected every hour for the first 9 h and further regularly checked after 21 h. BPA‐PC depolymerization was stable for 6 h using DMAP@PS in THF (60 °C) afterwards, the efficiency of the catalytic system started to drop, likely for the same reason as observed for the recycling under batch (**Figure** **4**a and Figure S18, Supporting Information). Under continuous flow, in analogy to batch experiments, the catalyst could be regenerated by treatment with a 6 M KOH solution in MeOH. However, a decline in catalyst activity was observed after 5 h (Figure S19, Supporting Information). PLA depolymerization in the presence of DMAP@PS in THF (60 °C) instead showed higher stability up to 9 h, while overnight, the catalyst activity dropped (Figure 4b and Figure S20, Supporting Information).

Finally, the developed catalytic system was tested for polymer waste recycling *via* glycolysis. Safety spectacles made of BPA‐PC and straws made of PLA were subjected to depolymerization under continuous flow and heterogeneous batch conditions. These small‐scale experiments were aimed at exploring the potential of applying such chemical recycling protocol to real waste plastics. The use of heterogeneous catalysts both under heterogeneous batch conditions and continuous flow showed full BPA‐PC conversion toward BPA and ethylene carbonate (Figure 4c and Figure S21 and S23, Supporting Information). Similarly, PLA waste was fully depolymerized to alkyl lactate under both batch and continuous flow approaches (Figure 4d and Figure S22 and S24, Supporting Information).

## 3 Conclusion

In conclusion, we have explored the use of supported catalysts for the depolymerization of plastics using milder reaction conditions (up to 60 °C). The use of a low boiling point solvent and milder reaction conditions aid in the product's recovery. Additionally, the application of supported organocatalysts enabled continuous flow chemical recycling of BPA‐PC and PLA, offering advantages such as extended catalyst stability and simplified process scale‐up. Finally, the established protocol was evaluated using real waste plastics.

This work represents the first example of plastic depolymerization under continuous flow using heterogeneous recyclable organocatalysts. The results achieved within this study pave the way for the design of one‐pot depolymerization and subsequent re‐polymerization under continuous flow to achieve a closed‐loop recycling process.

## Conflict of Interest

The authors declare no conflict of interest.

## Supporting information

Supplementary Material

## Data Availability

The data that support the findings of this study are available in the supplementary material of this article.
