# Supplementary material for: Continuous Flow Depolymerization of Polycarbonates and Poly(lactic acid) Promoted by Supported Organocatalysts
Source: ChemSusChem. 2025 Apr 25;18(13):e202500420. doi: 10.1002/cssc.202500420 (PMC12232091; doi:10.1002/cssc.202500420)
Supplement: Supplementary file 1 — Supplementary Material [file CSSC-18-e202500420-s001.pdf]

Supporting information

Continuous Flow Depolymerization of Polycarbonates and Poly(lactic acid) Promoted by Supported Organocatalysts

Madeleine Edge,[a] Neha Yadav,[a] Ali Al Rida Hmayed,[a] Andrew P. Dove,[a]* and Arianna Brandolese[a]*

[a]School of Chemistry, University of Birmingham, Edgbaston, Birmingham, B15 2TT (UK)

*Corresponding author email: a.brandolese@bham.ac.uk

List of abbreviations

2-HEtLa	2-Hydroxyethyl lactate
BPA	Bisphenol A
BPA-PC	Bisphenol A-based polycarbonate
DBU	1,8-Diazabicyclo[5.4.0]undec-7-ene
DMAP	4-Dimethylamino pyridine
DMSO	Dimethyl sulfoxide
EC	Ethylene carbonate
EG	Ethylene glycol
M_n	Number average molecular weight
M_w	Weight average molecular weight
NMR	Nuclear magnetic resonance
PLA	Poly(lactic acid)
PVP	Polyvinylpyridine
SEC	Size exclusion chromatography
TBD	1,5,7-Triazabicyclo[4.4.0]dec-5-ene

Table of contents

General Information	S4
Set-up Continuous Flow Depolymerization	S5
Solubility of Poly(bisphenol A carbonate) and Poly(lactic acid)	S6
General Procedure for Depolymerization Under Batch Conditions	S7
Catalyst Recycling Under Batch Conditions	S13
Catalyst Regeneration Under Batch Conditions	S17
Kinetic Studies Under Batch Conditions	S19
General Procedure for Depolymerization Under Continuous Flow Conditions	S20
Continuous Flow Long-run Experiments	S22
Waste Plastic Depolymerization	S25
References	S29

General Information

Materials

All chemicals were used without further purification. PLA and BPA-PC were purchased from Goodfellow Cambridge Limited. DBU polymer-bound (catalyst loading = 1.5-2.5 mol/g), DMAP polymer-bound (catalyst loading = ~3.0 mmol/g), Novozym® 435, TBD polymer-bound (catalyst loading = ~3 mmol/g), PVP, chloroform, cyrene, dichloromethane, dimethylformamide, and tetrahydrofuran were all purchased from Sigma Aldrich. Ethylene glycol was purchased from Honeywell.

Instruments

NMR Spectroscopy

A Bruker DPX-400 was used to record ^{1}H (400 MHz) spectra at 298 K. (Chemical shifts are reported as δ in parts per million (ppm), $CHCl_3$: ^{1}H δ = 7.26 ppm) and referenced to the chemical shift of the residual solvent resonances. The resonance multiplicities are described as s (singlet), d (doublet), t (triplet), q (quartet), dd (doublet of doublets) or m (multiplet).

Size Exclusion Chromatography (SEC)

Agilent 1260 Infinity II Multi-Detector GPC/SEC System was used for SEC measurements in $CHCl_3$ fitted with viscometer, ultraviolet (UV, λ = 309 nm), and infrared detectors. The reaction products were eluted in a $CHCl_3$ (in the presence of 0.5% NEt_3) mobile phase through an Agilent guard column (PLGel 5 µM, 50 × 7.5 mm) and two Agilent mixed-C columns (PLGel 5 µM, 300 × 7.5 mm) (40 °C, flow rate = 1 mL min^{-1}). Using Agilent GPC/SEC software (vA.02.01), number average molecular weights (M_n), weight average molecular weights (M_w) and dispersities ($Đ_M = M_w/M_n$) could be determined against a 15-point calibration curve (M_p = 162–3,187,000 g mol^{-1}) based on poly(styrene) standards (Easivial PS-M/H, Agilent).

Set-up Continuous Flow Depolymerization

Depolymerization reactions in continuous flow were completed using an EZ Omnifit® glass chromatography column 6.6 mm x 150 mm, 900 psi, with one adjustable end piece to adjust bed height. A heating tape was used to heat the column to perform the reaction at 60 ºC and 68 ºC (a thermometer was placed inside the column to measure the actual temperature at which the reaction was performed). Plastic tubing was used for maximum flexibility. An AL-300 syringe pump from World Precision Instruments was fitted with a 20 mL (inner diameter 20.1 mm) plastic syringe dispensing at a flow rate of 0.05-0.1 mL min^{-1}. At higher temperatures, a BPR (15 psi or 40 psi) was used. Residence time (τ) was determined using methyl red solution.

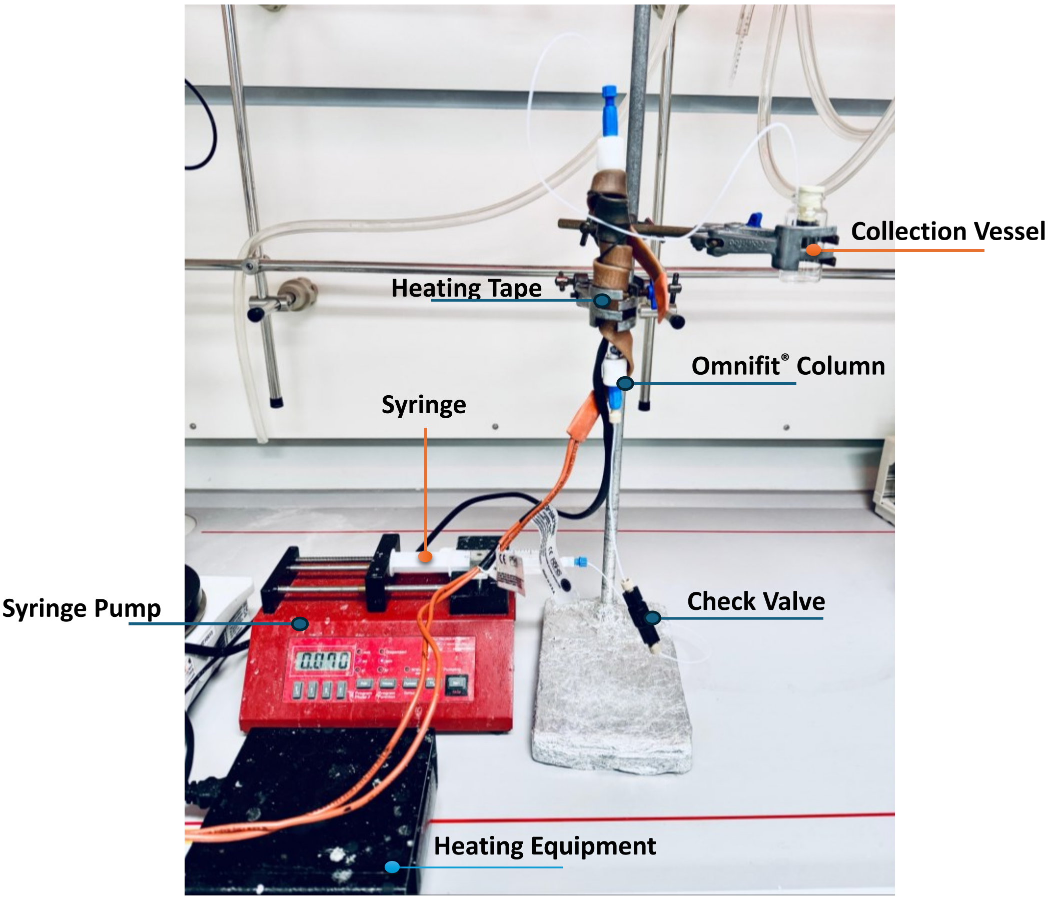

Figure S1. Continuous flow equipment set up with heating tape and heating equipment, AL-300 syringe pump fitted with a 20 mL plastic syringe and Onmifit® glass chromatography column.

Solubility of Poly(bisphenol A carbonate) and Poly(lactic acid)

The continuous flow approach requires a homogeneous solution: approximately 40 mg of PLA (1 pellet) or 15 mg of BPA-PC (1 pellet) were placed into a 20 mL vial and dissolved in 1 mL of chosen solvent. If dissolution had not occurred within 30 min, the vial was then heated to 40, 60 or 100 °C depending on the solvent's boiling point. This was left until dissolution occurred or for a maximum of 3 hours. This was then used to calculate the equivalent solvent per monomer unit for use in further reactions.

Table S1. Solubility screening of PLA and BPA-PC in a variety of solvents and solvent combinations. **Ins**: the polymer was not soluble in this solvent and temperature combination, **S**: the polymer was soluble in this solvent and temperature combination, blank - the combination was not tested.

**Solvent**	**BPA-PC**[a]				**PLA**[b]			
	**RT**	**40**	**60**	**80**	**RT**	**40**	**60**	**80**
Acetonitrile	Ins	Ins	Ins	-	Ins	Ins	Ins	-
Chloroform	S	-	-	-	S	-	-	-
Cyrene	Ins	Ins	Ins	S	Ins	Ins	Ins	S
Dichloromethane	S	-	-	-	S	-	-	-
Diethyl Carbonate	Ins	Ins	Ins	Ins	Ins	Ins	Ins	Ins
Dimethylformamide	S	-	-	-	Ins	-	S	S
Dimethyl Carbonate	Ins	Ins	Ins	Ins	-	S	S	S
Dimethyl Sulfoxide	Ins	-	-	S	Ins	-	-	S
Ethylene Glycol	Ins	Ins	Ins	Ins	Ins	Ins	Ins	Ins
Ethyl Acetate	Ins	Ins	Ins	-	Ins	Ins	Ins	-
Methanol	Ins	Ins	Ins	-	Ins	Ins	Ins	-
Methyl Ethyl Ketone	Ins	Ins	Ins	-	Ins	Ins	Ins	-
Methyl Tetrahydrofuran	Ins	Ins	Ins	-	Ins	Ins	Ins	-
Tetrahydrofuran	S	-	-	-	S	-	-	-
Toluene	Ins	Ins	Ins	Ins	Ins	Ins	Ins	S
Ethylene Glycol and Methyl Tetrahydrofuran	Ins	Ins	Ins	Ins	Ins	Ins	Ins	Ins

[a]BPA-PC concentration = 58-72 mM ; [b]PLA concentration = 480-610 mM

General Procedure for Depolymerization Under Batch Conditions

Poly(lactic acid) Depolymerization

Pellets of PLA (80 mg, 1.13 mmol, 1.0 equiv.) were loaded in a 20 mL vial and dissolved in the stated solvent (0.12 M). Ethylene glycol (0.63 mL, 11 mmol, 10 equiv.) and the supported catalyst (20 mol%) were subsequently added after dissolution as well as an oval magnetic stirrer. The mixture was transferred to a pre-heated hot plate at the desired reaction temperature and was left stirring for 24 hours. The solvent was removed under vacuum, and the reaction mixture was diluted with DCM, and washed with brine. The organic layer was dried over $MgSO_4$, filtered and concentrated under reduced pressure to give 2- HEtLa as a colourless oil (136 mg, 90%) with spectroscopic data in accordance with the literature.[1] The conversion was monitored *via* ^{1}H NMR spectroscopy using aliquots taken at specified intervals or after the completion of the reaction. The depolymerization yield was calculated *via* the integration of characteristic PLA signals *i.e.,* δ (ppm) 1.59 (d, 3H) compared to characteristic alkyl lactate product (2-HEtLa) signals *i.e.,* δ (ppm) 1.45 (d, 3H) as previously reported in the literature.[1] Depolymerization was also confirmed using SEC.

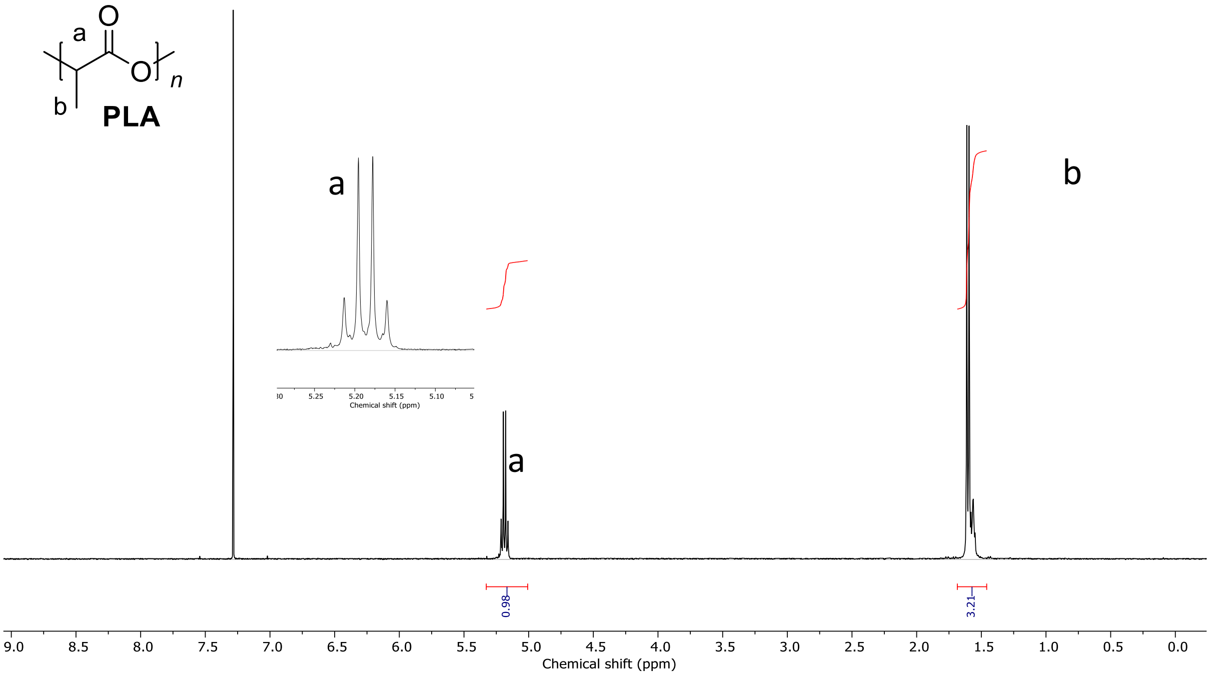

Figure S2. ^{1}H NMR spectrum of PLA.
^{1}H NMR (400 MHz, $CDCl_3$) δ_H = 5.16 (q, J = 7.1 Hz, 1H), 1.59 (d, J = 7.1 Hz, 3H).

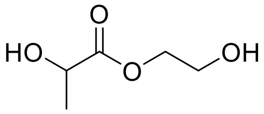

2-HEtLa

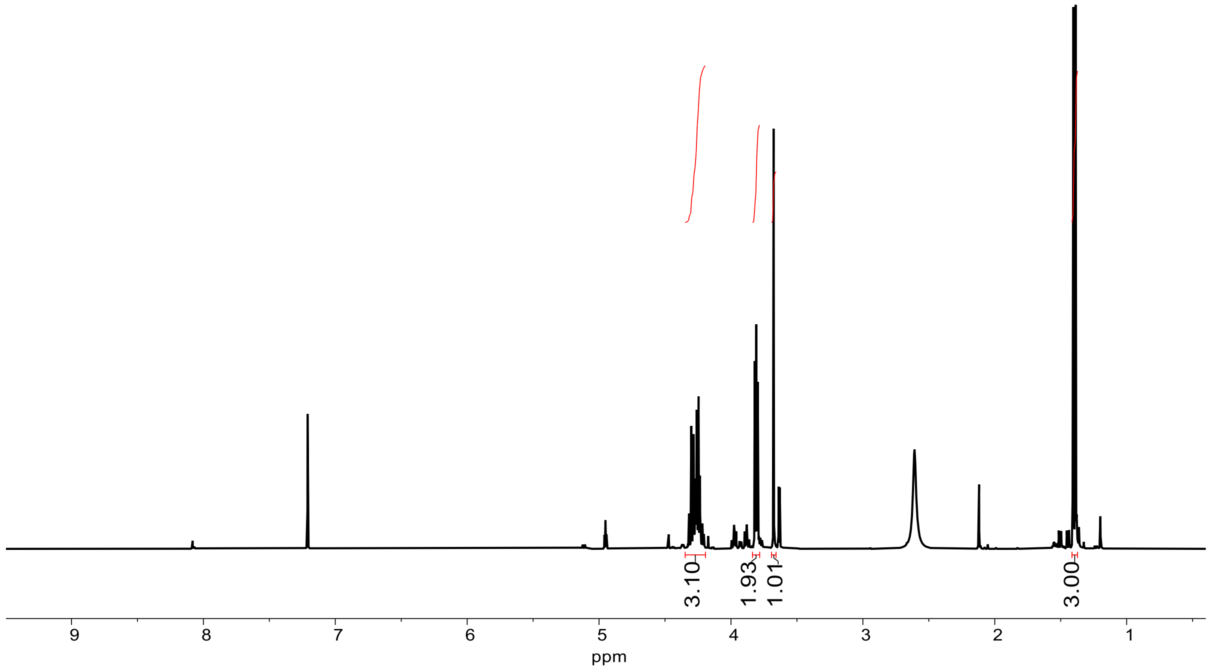

Figure S3. ^{1}H NMR spectrum of 2-HEtLa.
^{1}H NMR ($CDCl_3$, 400 MHz) δ_H = 4.35 – 4.19 (m, 3H), 3.81 (dd, J = 4.9, 4.3 Hz, 2H), 3.68 (s, 1H), 1.45 (d, J = 6.9 Hz, 3H).

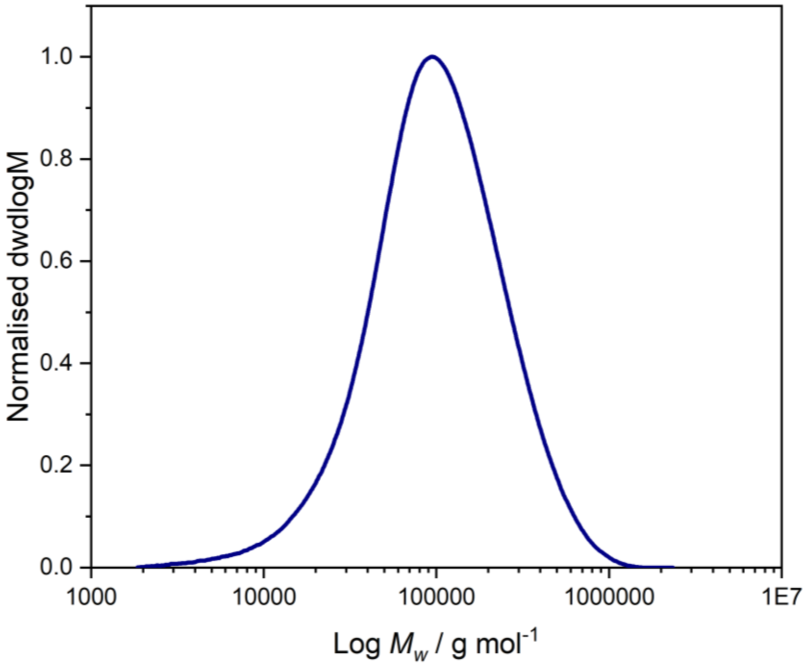

Figure S4. SEC chromatogram of PLA ($CHCl_3$ + 0.5% NEt_3) M_w = 141 kg mol^{-1}, M_n = 61.5 kg mol^{-1}, M_p = 96.2 kg mol^{-1}, $Đ_M$ = 2.29.

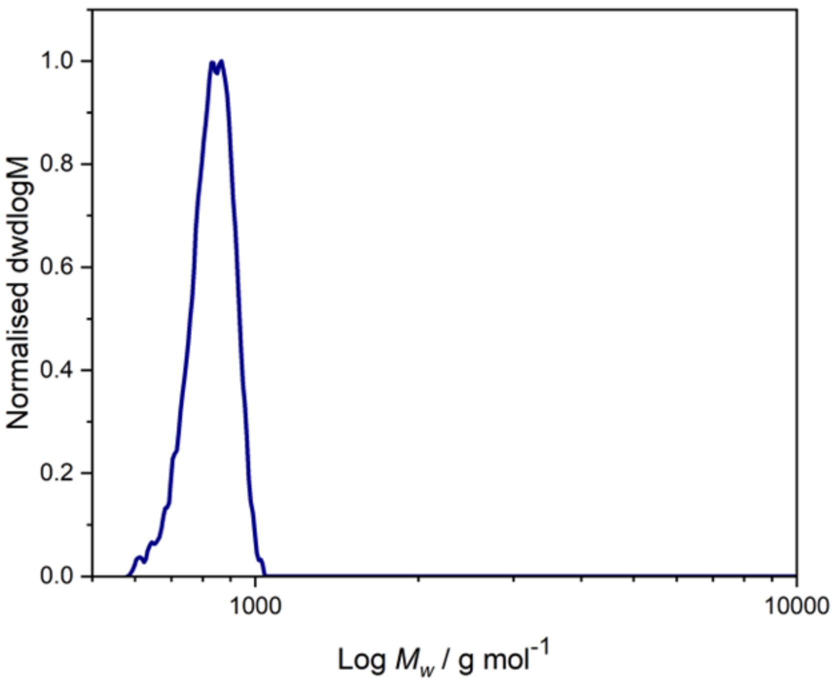

Figure S5. SEC chromatogram of PLA depolymerization product ($CHCl_3$ + 0.5% NEt_3) M_w = 863 g mol^{-1}, M_n = 826 g mol^{-1}, M_p = 866 g mol^{-1}, $Đ_M$ = 1.04.

Poly(bisphenol A carbonate) Depolymerization

Pellets of BPA-PC (70 mg, 0.27 mmol, 1.0 equiv.) were loaded in a 20 mL vial and dissolved in the stated solvent (0.12 M). Ethylene glycol (0.15 mL, 2.7 mmol, 10 equiv.) and catalyst (20 mol%) were subsequently added after dissolution as well as an oval magnetic stirrer. The mixture was transferred to a pre-heated hot plate at the desired reaction temperature and was left stirring for 24 hours. The solvent was removed under vacuum, and the reaction mixture was diluted with DCM, and washed with brine. The organic layer was dried over $MgSO_4$, filtered and concentrated under reduced pressure. The crude product was purified by column chromatography (60:40 hexane: ethyl acetate) to give BPA as a white solid (57 mg, 93%) and ethylene carbonate as a white solid (22 mg, 92%) with spectroscopic data in accordance with the literature.[2] The conversion was monitored *via* ^{1}H NMR spectroscopy using aliquots taken at specified intervals or after the completion of the reaction. The depolymerization yield was calculated *via* the integration of characteristic BPA-PC signals *i.e.*, δ (ppm) 7.26 (d, 4H) and 7.18 (d, 4H) compared to characteristic BPA product signals *i.e.*, δ (ppm) 7.10 (d, 4H) and 6.74 (d, 4H) as previously reported in the literature.[2] Depolymerization was also confirmed using SEC.

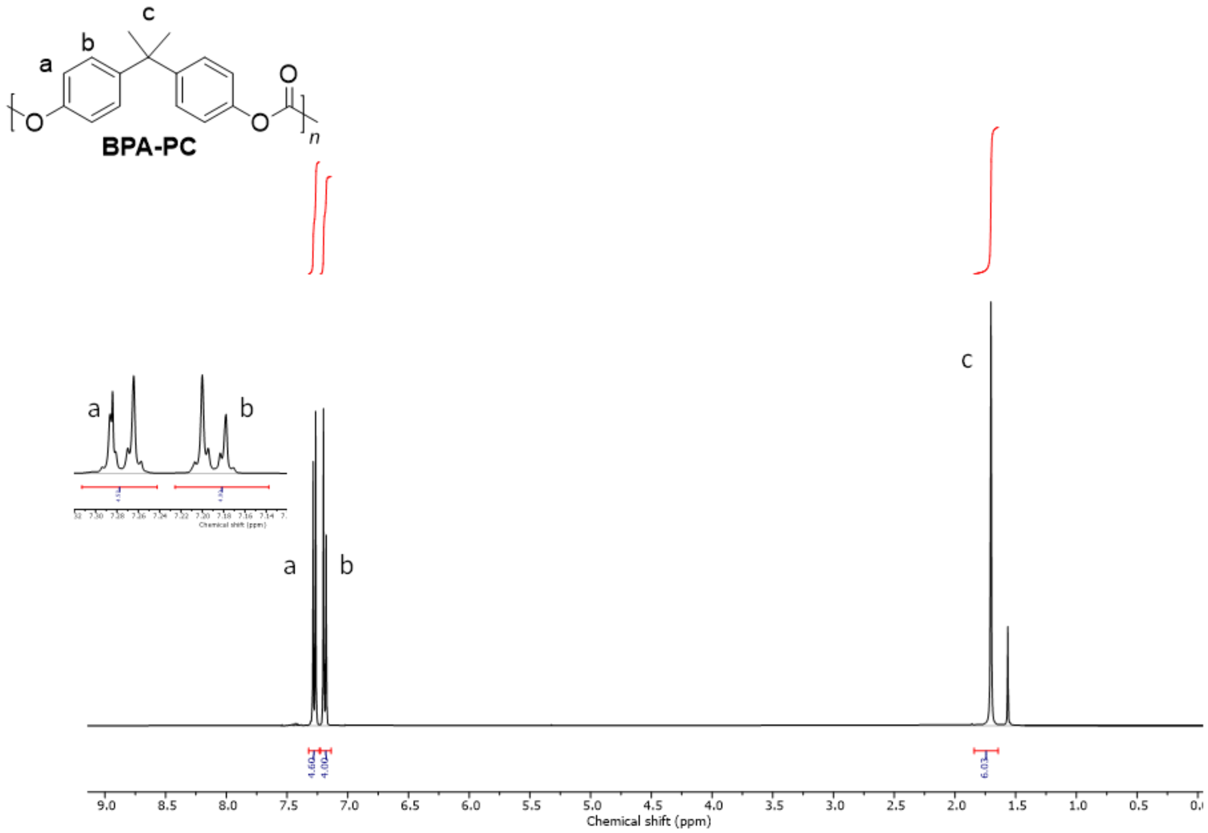

Figure S6. ^{1}H NMR spectrum of BPA-PC.

^{1}H NMR (400 MHz, Chloroform-*d*) δ_H= 7.26 (d, J = 7.9 Hz, 4H), 7.18 (d, J = 8.9 Hz, 4H), 1.68 (s, 6H).

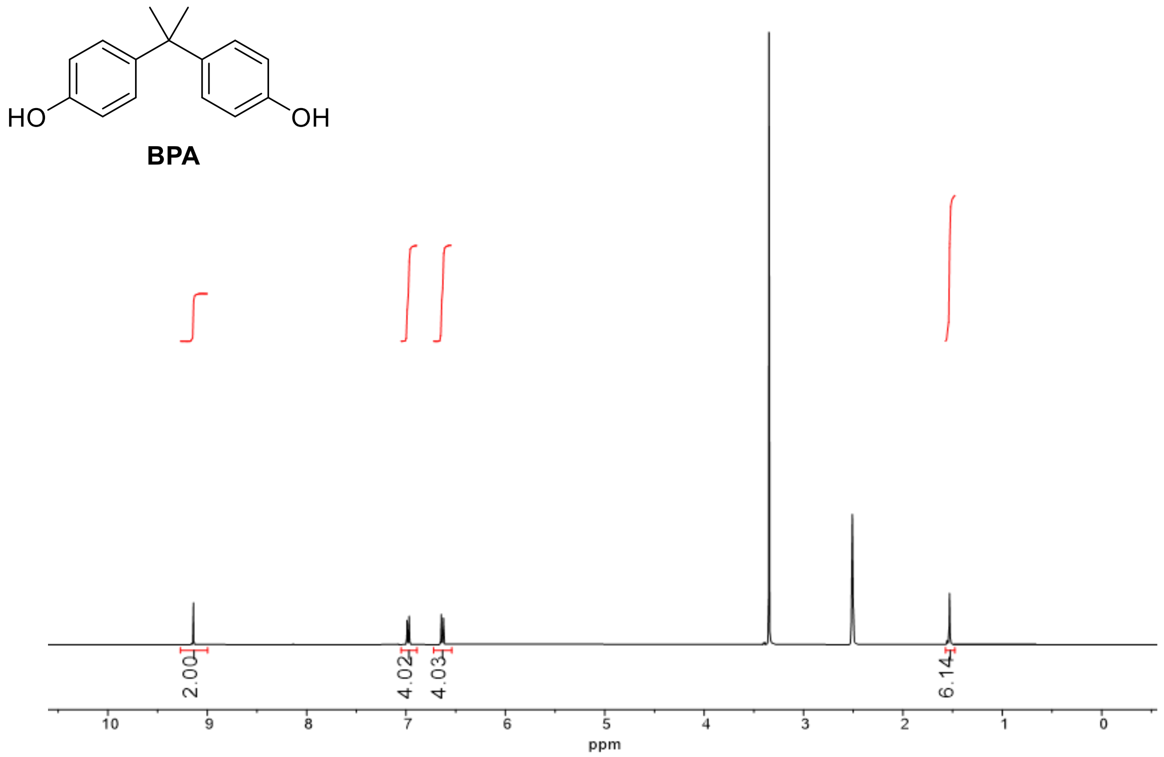

Figure S7. ^{1}H NMR spectra of the isolated BPA.
^{1}H NMR (DMSO-d_6, 400 MHz) δ_H = 9.14 (s, 2H), 7.05 – 6.89 (m, 4H), 6.72 – 6.54 (m, 4H), 1.53 (s, 6H).

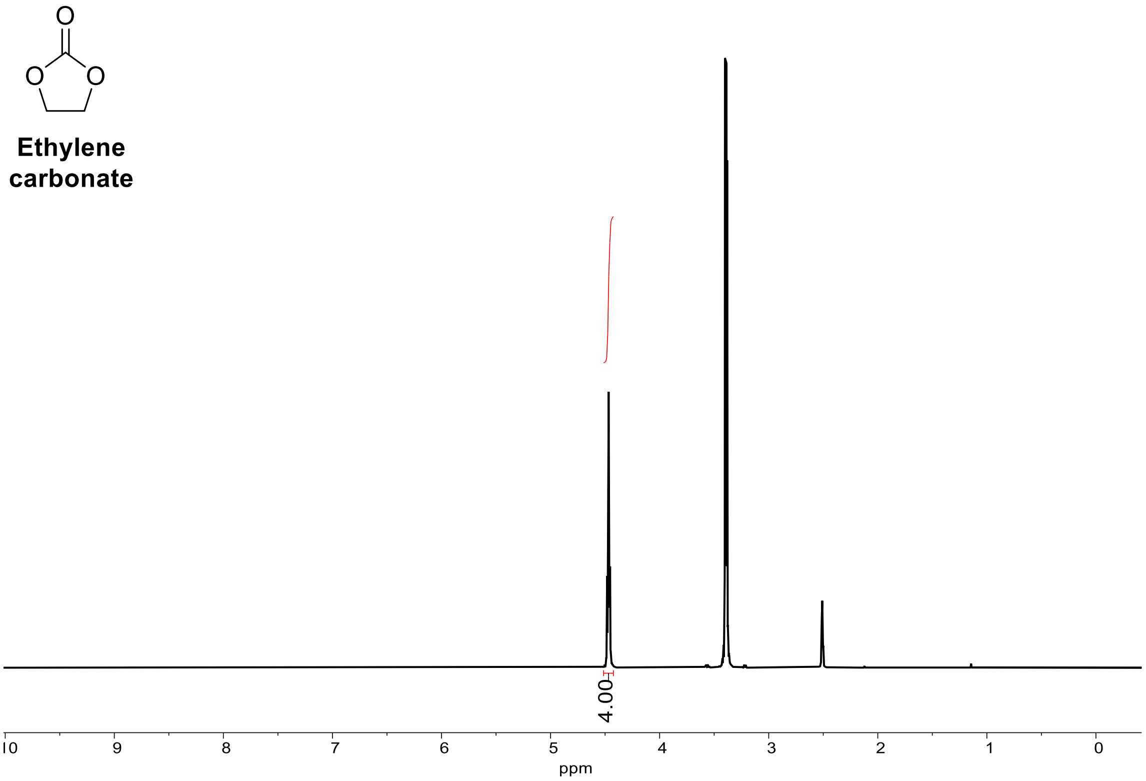

Figure S8. ^{1}H NMR spectra of the isolated ethylene carbonate.
^{1}H NMR (DMSO-d_6, 400 MHz) δ_H = 4.51 – 4.42 (m, 4H).

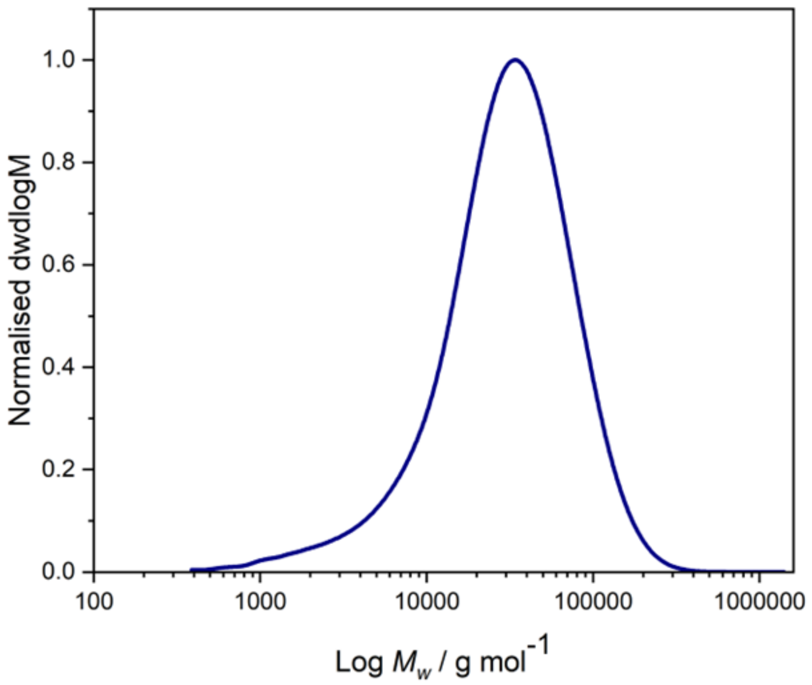

Figure S9. SEC chromatogram of BPA-PC ($CHCl_3$ + 0.5% NEt_3) M_w = 42.1 kg mol^{-1}, M_n = 15.8 kg mol^{-1}, M_p = 34.7 kg mol^{-1}, $Đ_M$ = 2.67.

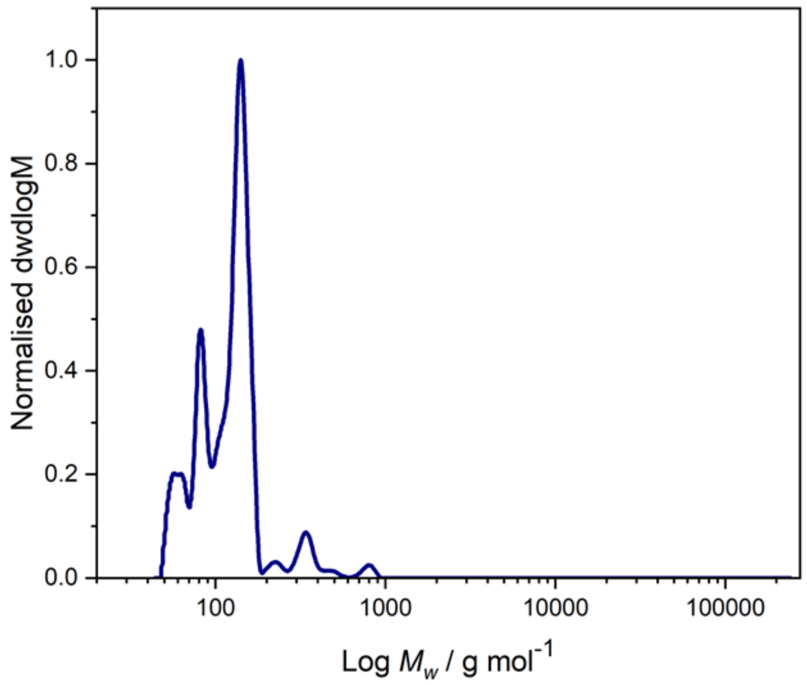

Figure S10. SEC chromatogram of BPA-PC depolymerization product ($CHCl_3$ + 0.5% NEt_3) M_w = 136 g mol^{-1}, M_n = 109 g mol^{-1}, M_p = 141 g mol^{-1}, $Đ_M$ = 1.25.

Catalyst Recycling under Batch Conditions

Batch reactions for each polymer were repeated for up to 5 cycles using both DBU@PS and DMAP@PS as catalysts. After 24 hours, once the reaction was complete, the catalyst was filtered from the reaction mixture, washed with the corresponding reaction solvent (PLA: DMF for DMAP@PS and THF for DBU@PS; BPA-PC: DMF for both catalysts) to remove any residual depolymerization products, and subsequently dried under vacuum for 5 h and weighed. The recovered catalyst was then introduced into a fresh batch of dissolved polymer and alcohol, to perform the following depolymerization cycle following the general protocol.

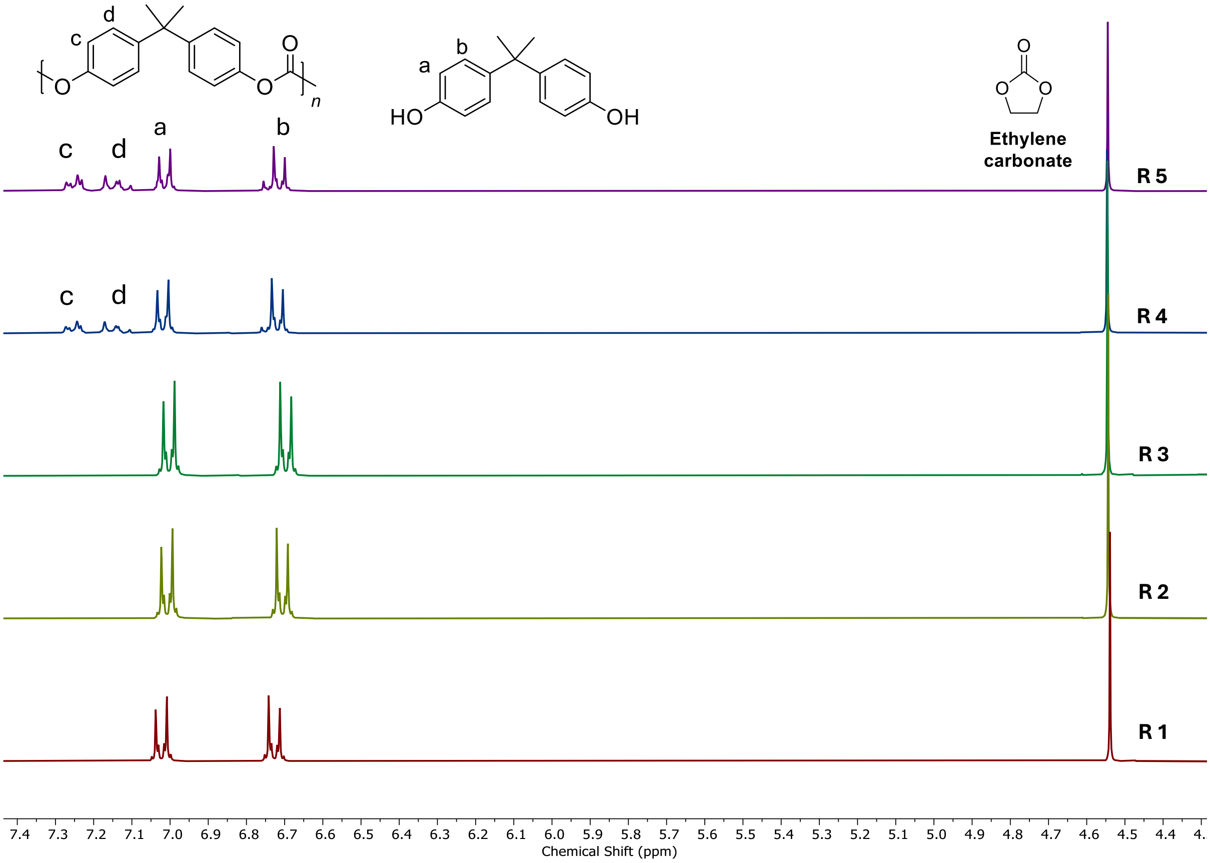

Figure S11. Stacked ^{1}H NMR ($CHCl_3$, 400 MHz) spectra of BPA-PC depolymerization with EG (10 equiv.), DBU@PS (20 mol%) in DMF at 80 °C in batch showing catalyst recycling (R1 – R5).

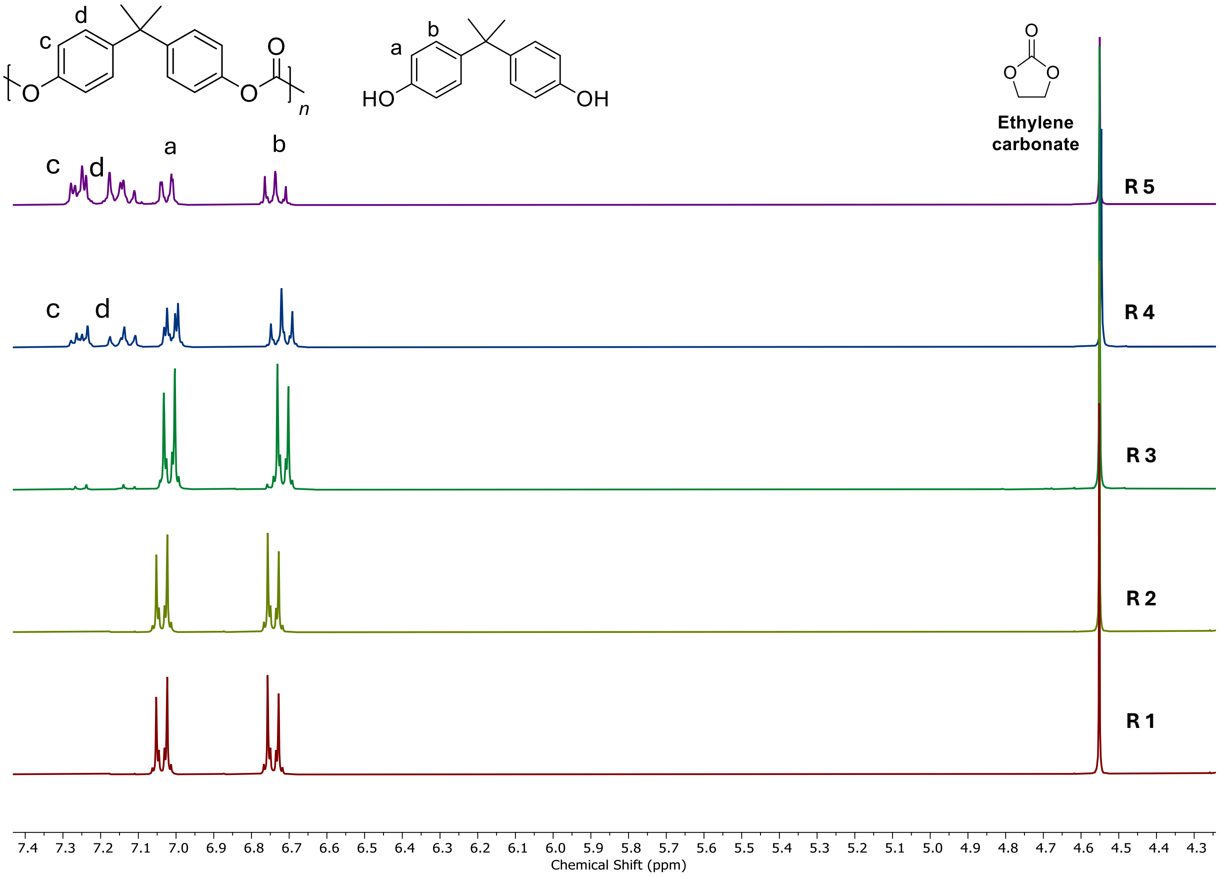

Figure S12. Stacked ^{1}H NMR ($CHCl_3$, 400 MHz) spectra of BPA-PC depolymerization with EG (10 equiv.), DMAP@PS (20 mol%) in DMF at 80 °C in batch showing catalyst recycling (R1 – R5).

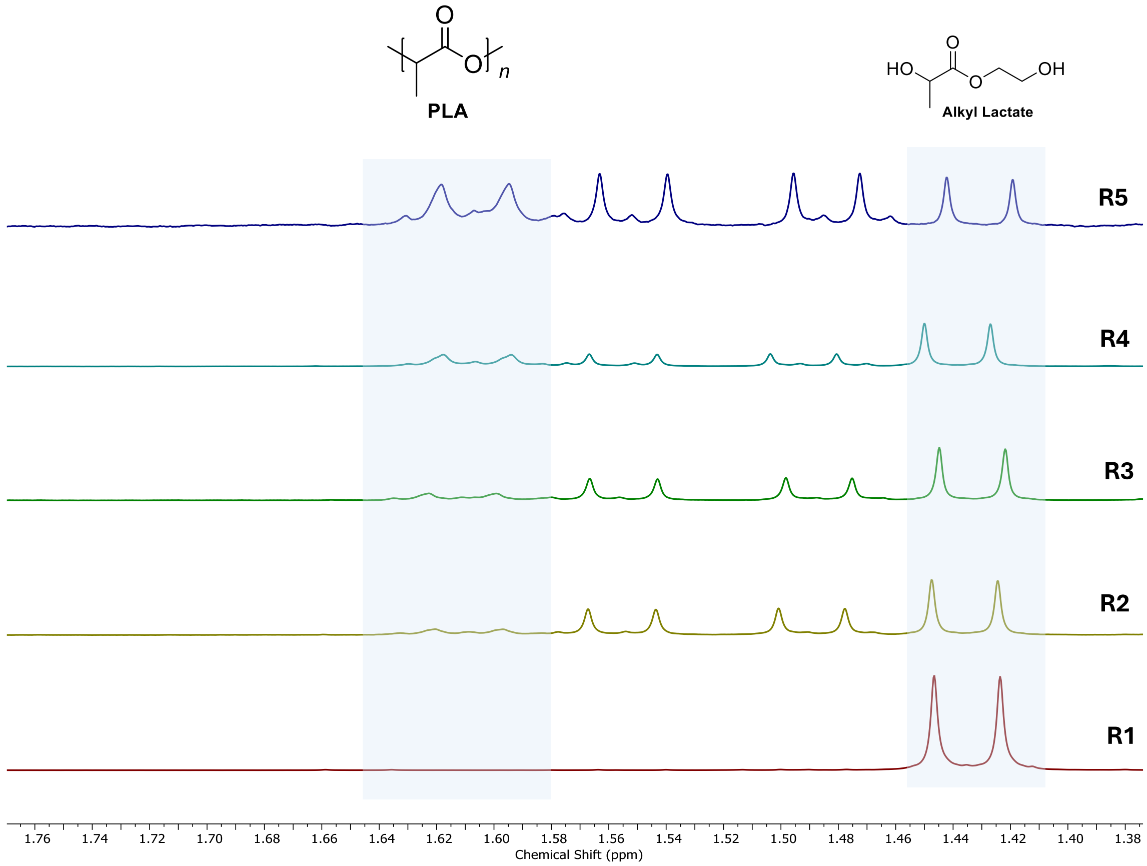

Figure S13. Stacked ^{1}H NMR ($CHCl_3$, 400 MHz) spectra of PLA depolymerization with EG (10 equiv.), DBU@PS (20 mol%) in THF at 60 °C in batch showing catalyst recycling (R1 – R5).

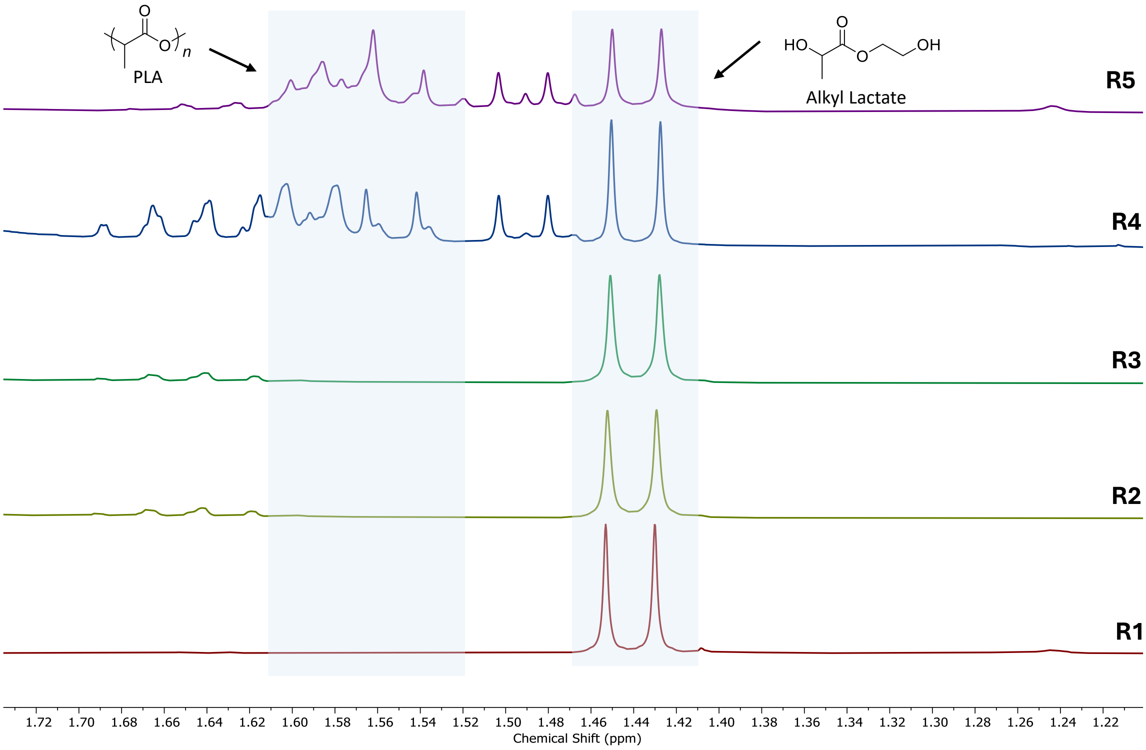

Figure S14. Stacked ^{1}H NMR ($CHCl_3$, 400 MHz) spectra of PLA depolymerization with EG (10 equiv.), DMAP@PS (20 mol%) in DMF at 80 °C in batch showing catalyst recycling (R1 – R5).

Catalyst Regeneration Under Batch Conditions

Partial catalyst deactivation was observed after a couple of recycles; therefore, after 5 cycles the catalyst was regenerated. Both DMAP@PS and DBU@PS were washed with a 6 M methanolic KOH solution for 2 h, followed by three washes with methanol to ensure complete removal of KOH. The regenerated catalyst was then dried, weighed, and reused for the depolymerization of a fresh batch of dissolved polymer and ethylene glycol. The reaction was conducted for 16 hours, and conversion was monitored *via* ^{1}H-NMR spectroscopy.

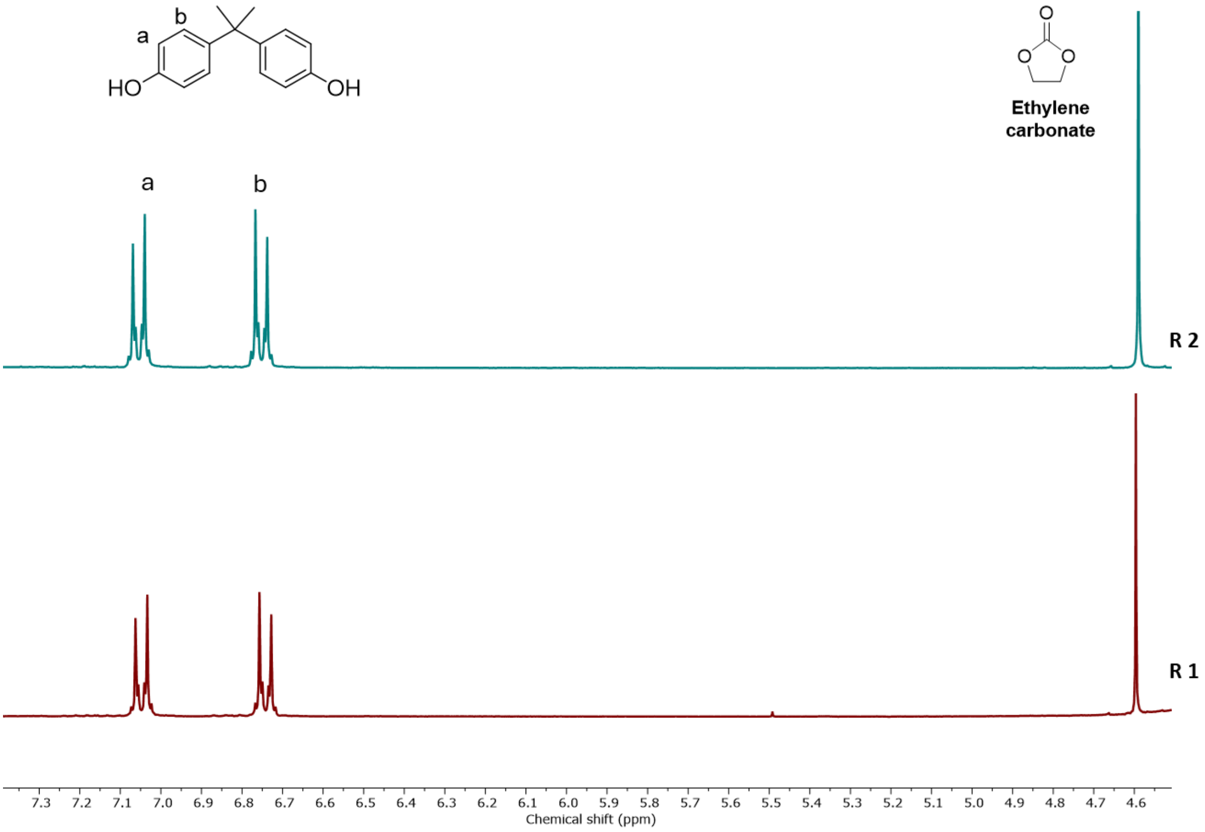

Figure S15. ^{1}H NMR ($CHCl_3$, 400 MHz) spectrum of BPA-PC depolymerization under batch using regenerated DBU@PS (20 mol%) and EG (10 equiv.) in DMF at 80 ºC. R1 = first depolymerization cycle after regeneration; R2 = second depolymerization cycle after regeneration.

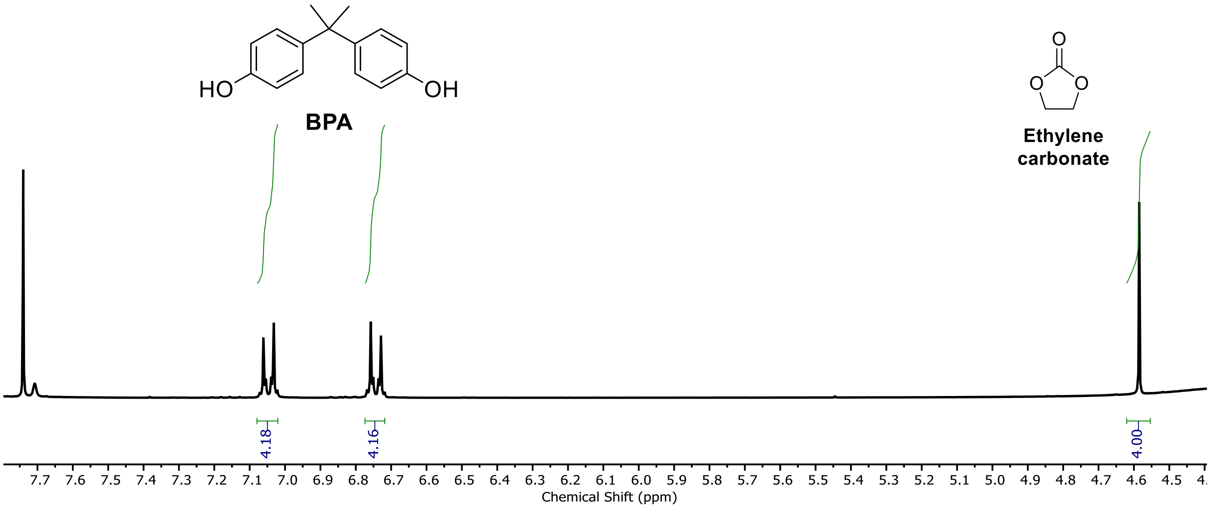

Figure S16. ^{1}H NMR ($CHCl_3$, 400 MHz) spectrum of BPA-PC depolymerization under batch using regenerated DMAP@PS (20 mol%) and EG (10 equiv.) in DMF at 80 ºC.

Kinetic Studies Under Batch Conditions

Kinetic studies were carried out following the general protocols reported above. Aliquots were taken every hour and analyzed *via* ^{1}H NMR spectroscopy.

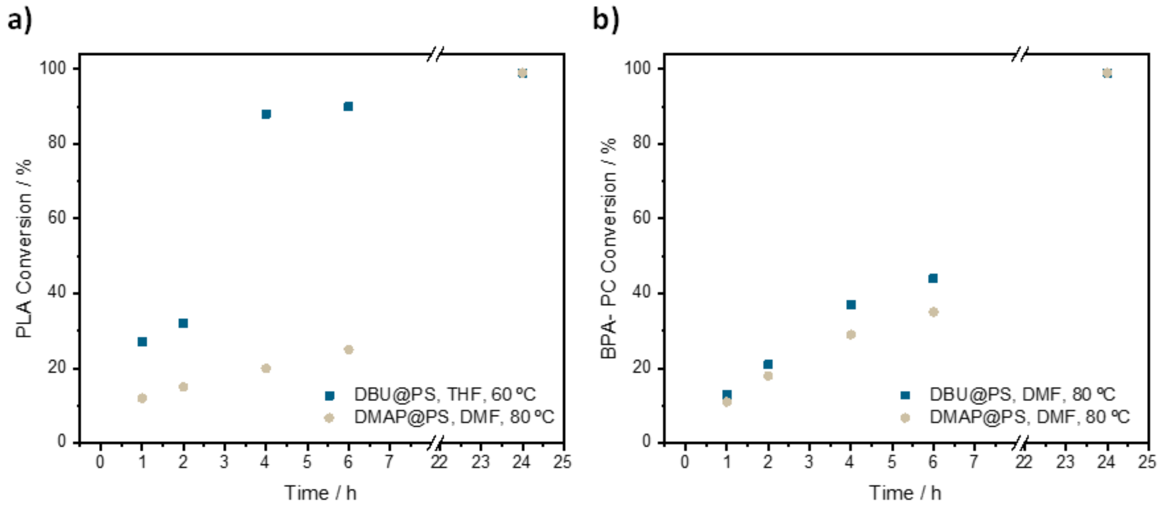

Figure S17. Kinetic plots for (a) PLA depolymerization with DBU@PS in THF at 60 ºC (blue squares) and DMAP@PS in DMF at 80 ºC (grey dots). Alkyl lactate yield was calculated *via* the integration of characteristic PLA signals *i.e., δ* (ppm) 1.59 (d, 3H) compared to characteristic alkyl lactate product signals *i.e., δ* (ppm) 1.45 (d, 3H). (b) BPA-PC depolymerization with DBU@PS in DMF at 80 ºC (blue squares) and DMAP@PS in DMF at 80 ºC (grey dots). BPA yield was calculated *via* the integration of characteristic BPA-PC signals *i.e., δ* (ppm) 7.26 (d, 4H) and 7.18 (d, 4H) compared to characteristic BPA product signals *i.e., δ* (ppm) 7.10 (d, 4H) and 6.74 (d, 4H).

General Procedure for Depolymerization Under Continuous Flow Conditions

Procedure for Poly(lactic acid) Depolymerization

The catalyst (0.7 g) was weighed and added to the reaction column. The stated solvent (THF or DMF) was placed in a syringe and passed through the column at the desired flow rate until no air remained. The catalyst was allowed to swell and then a measurement of the column length was taken. If heating was used, the system was heated to 60 °C using a heating tape (or 68 ºC for DMF), keeping a continuous flow of the solvent within the system. Pellets of PLA (160 mg, 2.25 mmol, 1.0 equiv.) were dissolved in the solvent (14.9 mL). Ethylene glycol (1.3 mL, 22.5 mmol, 10 equiv.) was then added to this solution. This was then passed through the column at the desired flow rate. Products were collected every 1 hour in 20 mL vials.
The solvent was then removed under vacuum, and the reaction mixture was diluted with DCM and washed with brine. The organic layer was dried over $MgSO_4$, filtered and concentrated under reduced pressure to give 2-HEtLa as a colourless oil with spectroscopic data in accordance with the literature.[1] The reaction was monitored *via* ^{1}H NMR spectroscopy by taking aliquots from each fraction. The depolymerization yield was calculated *via* the integration of characteristic PLA signals *i.e.,* δ (ppm) 1.59 (d, 3H), compared to characteristic alkyl lactate product signals *i.e.,* δ (ppm) 1.45 (d, 3H).

Procedure for Poly(bisphenol A carbonate) Depolymerization

The catalyst (0.7 g) was weighed and added to the reaction column. The stated solvent (THF or DMF) was placed in a syringe and passed through the column at the desired flow rate until no air remained. The catalyst was allowed to swell and then a measurement of the column length was taken. If heating was used, the system was heated to 60 °C using heating tape, keeping a continuous flow of solvent within the system. Pellets of BPA-PC (210 mg, 0.82 mmol, 1.0 equiv.) were dissolved in the solvent (14 mL). Ethylene glycol (0.5 mL 8.2 mmol, 10 equiv.) was then added to this solution. This was then passed through the column at the desired flow rate. Products were collected every 1 hour in 20 mL vials. The solvent was removed under vacuum, and the reaction mixture was diluted with DCM, and washed with brine. The organic layer was dried over $MgSO_4$, filtered and concentrated under reduced pressure. The reaction was monitored *via* ^{1}H NMR spectroscopy by taking aliquots from each fraction. The depolymerization yield was calculated *via* the integration of characteristic BPA-PC signals *i.e.,* δ (ppm) 7.26 (d, 4H) and 7.18 (d, 4H) compared to characteristic BPA product signals *i.e.,* δ (ppm) 7.10 (d, 4H) and 6.74 (d, 4H).

Table S2. Full reaction conditions screening for BPA-PC depolymerization under continuous flow conditions using supported catalysts.[a]

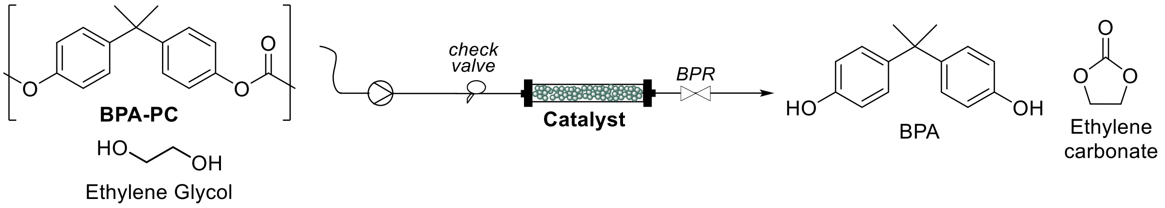

Entry	Catalyst	Solvent	T / °C	Flow rate / mL min^{-1}	τ / min	BPA / %[b]
1	DBU@PS	THF	rt	0.1	40	<5%
2	DBU@PS	THF	rt	0.05	80	<5%
3	DBU@PS	DMF	68	0.05	80	>99%
4[c]	DMAP@PS	THF	rt	0.1	40	56%
5	DMAP@PS	THF	rt	0.05	80	60%
6[c]	DMAP@PS	THF	60	0.05	80	>99%
7[c]	DMAP@PS	DMF	rt	0.1	40	55%
8[c]	DMAP@PS	DMF	rt	0.05	80	67%
9[c]	DMAP@PS	DMF	68	0.05	80	>99%

[a]Reaction conditions: BPA-PC (0.83 mmol, 1.0 equiv), EG (10 equiv), solvent (0.06 M), 0.7 g of catalyst (DBU@PS catalyst loading = 1.5-2.5 mol/g; DMAP@PS catalyst loading = ~3.0 mmol/g). [b]BPA NMR yield detected by ^{1}H NMR spectroscopy, measured when the steady-state regime was reached. [c]Amount of EG used = 30 equiv. τ stands for residence time.

Continuous Flow Long-run Experiments for BPA-PC Depolymerization

Following the general procedure for the BPA-PC depolymerization under continuous flow, the long-run experiment was conducted using an HPLC pump to feed the starting material. The solution of BPA-PC and ethylene glycol was injected at a flow rate of 0.05 mL min^{-1} and the crude was collected during the day (for 30 h) while analyzed by ^1H NMR ($CDCl_3$) spectroscopy. The depolymerization yield was calculated *via* the integration of characteristic BPA-PC signals *i.e., δ* (ppm) 7.26 (d, 4H) and 7.18 (d, 4H) compared to characteristic BPA product signals *i.e., δ* (ppm) 7.10 (d, 4H) and 6.74 (d, 4H).

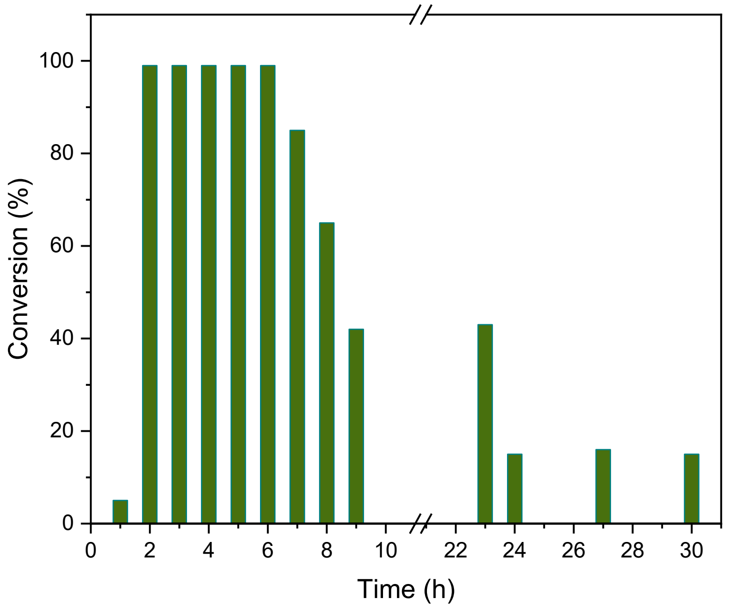

Figure S18. Study of catalyst stability under continuous conditions for BPA-PC depolymerization.

Catalyst Regeneration Under Continuous Flow Conditions

After 24 h long run experiment, the catalyst was washed for 3 h with a solution of 6 M KOH in MeOH at a flow rate of 0.100 mL/min. Afterwards, MeOH was flowed through the system for 1 h at 0.100 mL/min to ensure that all the KOH was removed, followed by THF for 1 h at 0.05 mL/min.. The solution of BPA-PC and ethylene glycol was thus injected at a flow rate of 0.05 mL min^{-1} and fractions was collected during the day (for 6 h) while analyzed by ^{1}H NMR ($CDCl_3$) spectroscopy. The depolymerization yield was calculated *via* the integration of characteristic BPA-PC signals *i.e., δ* (ppm) 7.26 (d, 4H) and 7.18 (d, 4H) compared to characteristic BPA product signals *i.e., δ* (ppm) 7.10 (d, 4H) and 6.74 (d, 4H).

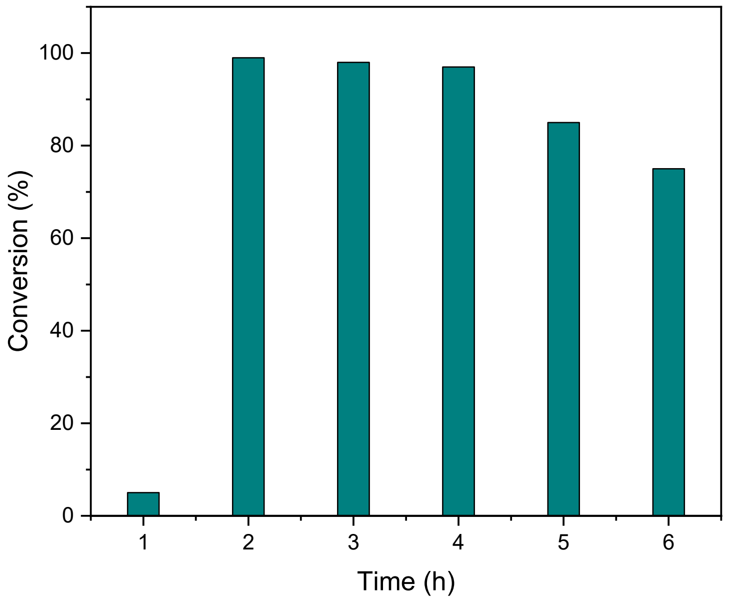

Figure S19. Study of catalyst stability after regeneration with KOH (6 M in MeOH) under continuous conditions for BPA-PC depolymerization.

Continuous Flow Long-run Experiments for PLA Depolymerization

Following the general procedure for the PLA depolymerization under continuous flow, the long-run experiment was conducted using an HPLC pump to feed the starting material. The solution of PLA and ethylene glycol was injected at a flow rate of 0.05 mL min^{-1} and the crude was collected during the day (for 30 h) while analyzed by ^{1}H NMR ($CDCl_3$) spectroscopy. The depolymerization yield was calculated *via* the integration of characteristic PLA signals *i.e., δ* (ppm) 1.59 (d, 3H), compared to characteristic alkyl lactate product signals *i.e., δ* (ppm) 1.45 (d, 3H).

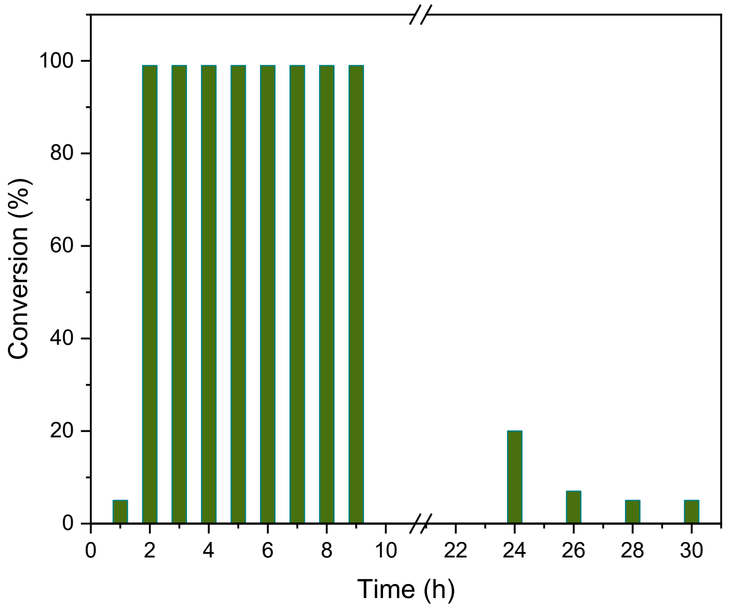

Figure S20. Study of catalyst stability under continuous conditions for PLA depolymerization.

Waste Plastic Depolymerization Under Batch Conditions

The developed catalytic system was tested for plastic waste recycling through glycolysis. BPA safety goggles and PLA straws were selected as plastic waste materials. PLA straws were cut into small pieces (80 mg, 1.13 mmol, 1.0 equiv.) and placed in a 20 mL vial, where they were dissolved in DMF (15 equiv.). After complete dissolution, ethylene glycol (0.63 mL, 11 mmol, 10 equiv.), catalyst DMAP@PS (20 mol%), and an oval magnetic stirrer were added. Similarly, BPA-PC goggles were broken into small pieces (70 mg) and dissolved in DMF (15 equiv.). Ethylene glycol (0.15 mL, 2.7 mmol, 10 equiv.) and catalyst DMAP@PS (20 mol%) were added after dissolution, and the reaction mixture was stirred at 80 °C. The conversion was monitored using ^{1}H NMR spectroscopy upon reaction completion. Depolymerization conversion was determined by analyzing the disappearance of characteristic BPA-PC signals at δ (ppm) 7.26 (d, 4H) and 7.18 (d, 4H), along with the appearance of BPA product signals at δ (ppm) 7.10 (d, 4H) and 6.74 (d, 4H). For PLA, depolymerization was assessed by comparing the PLA signal at δ (ppm) 1.59 (d, 3H) with the characteristic alkyl lactate product signal at δ (ppm) 1.45 (d, 3H).

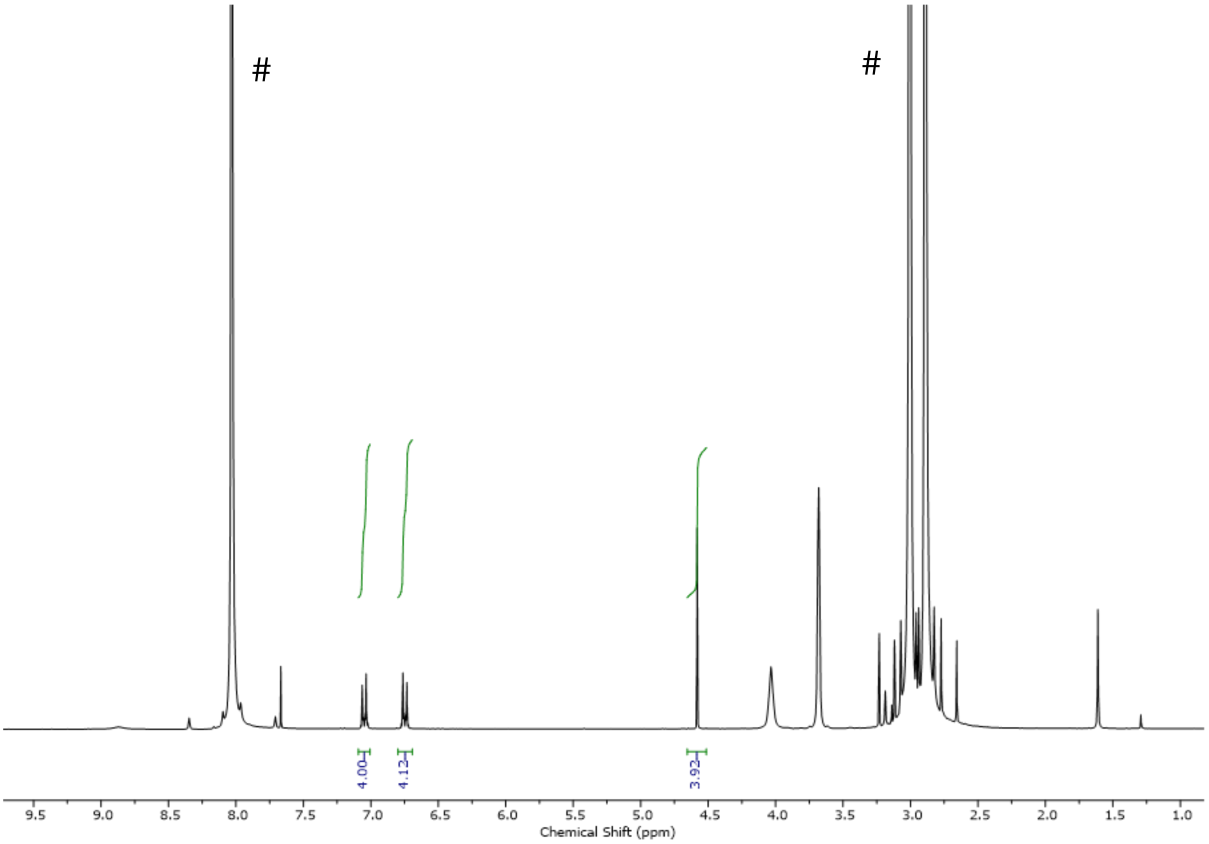

Figure S21. ^{1}H NMR ($CHCl_3$, 400 MHz) spectra of the reaction crude of the depolymerization of BPA-PC goggles waste under batch conditions. # = DMF.

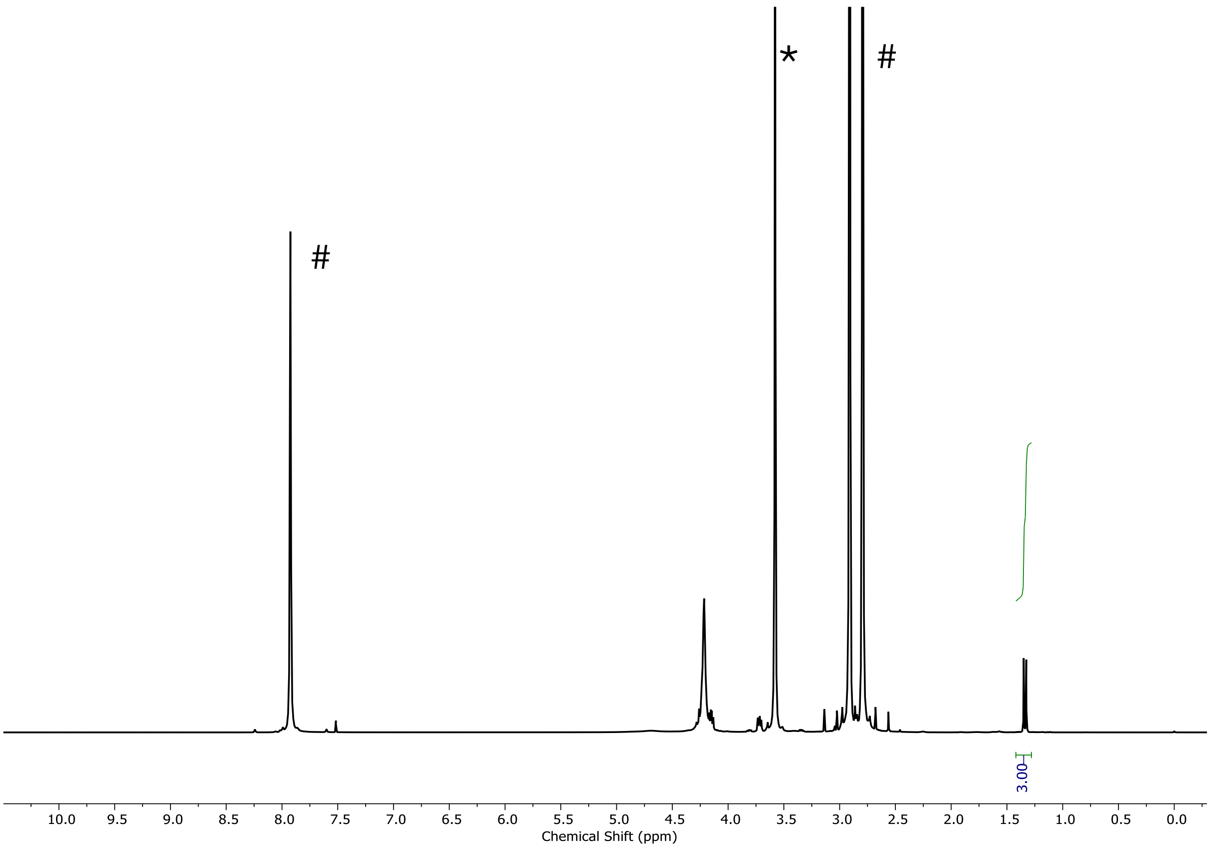

Figure S22. ^{1}H NMR ($CHCl_3$, 400 MHz) spectra of the reaction crude of the depolymerization of PLA straw waste under batch conditions. * = Ethylene Glycol; # = DMF.

Waste Plastic Depolymerization Under Continuous Flow

DMAP@PS (0.7 g) was weighed and added to the reaction column. THF was placed in a syringe and passed through the column at the desired flow rate until no air remained. The catalyst was allowed to swell and then a measurement of the column length was taken. The system was heated to 60 °C using heating tape, keeping a continuous flow of solvent within the system.

PLA straws were cut into small pieces (160 mg, 2.25 mmol, 1.0 equiv.) and dissolved in THF (14 mL). Ethylene glycol (0.5 mL, 8.2 mmol, 10 equiv.) was then added to this solution. This was then passed through the column at the desired flow rate. Products were collected every 1 hour in 20 mL vials until the reaction was completed. The reaction was monitored *via* ^{1}H NMR spectroscopy by taking aliquots from each fraction.

Similarly, BPA-PC goggles were broken into small pieces (210 mg, 0.82 mmol, 1.0 equiv.) and dissolved in THF (14 mL). Ethylene glycol (0.5 mL, 8.2 mmol, 10 equiv.) was then added to this solution. This was then passed through the column at the desired flow rate. Products were collected every 1 hour in 20 mL vials until the reaction was completed. The reaction was monitored *via* ^{1}H NMR spectroscopy by taking aliquots from each fraction.

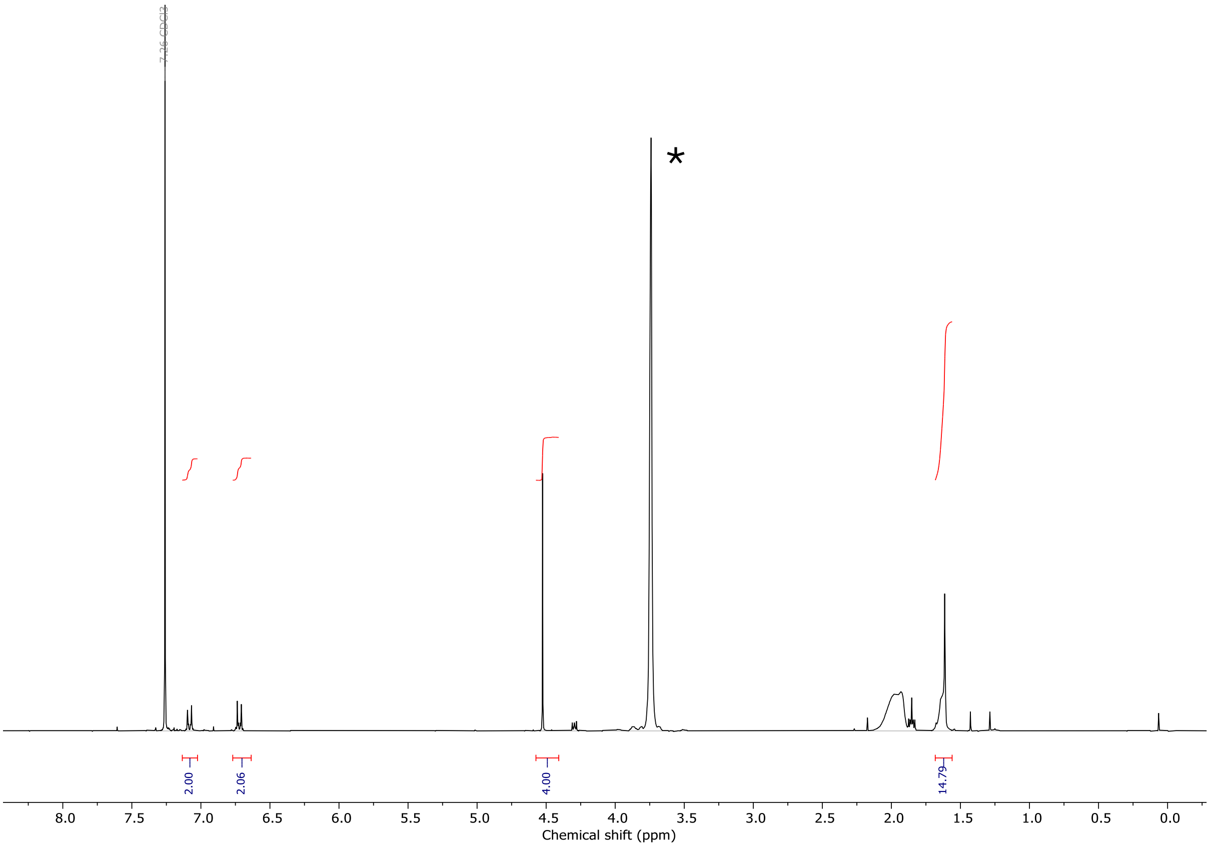

Figure S23. ^{1}H NMR ($CHCl_3$, 400 MHz) spectra of the reaction crude of the depolymerization of BPA-PC goggles waste under continuous flow. * = Ethylene Glycol.

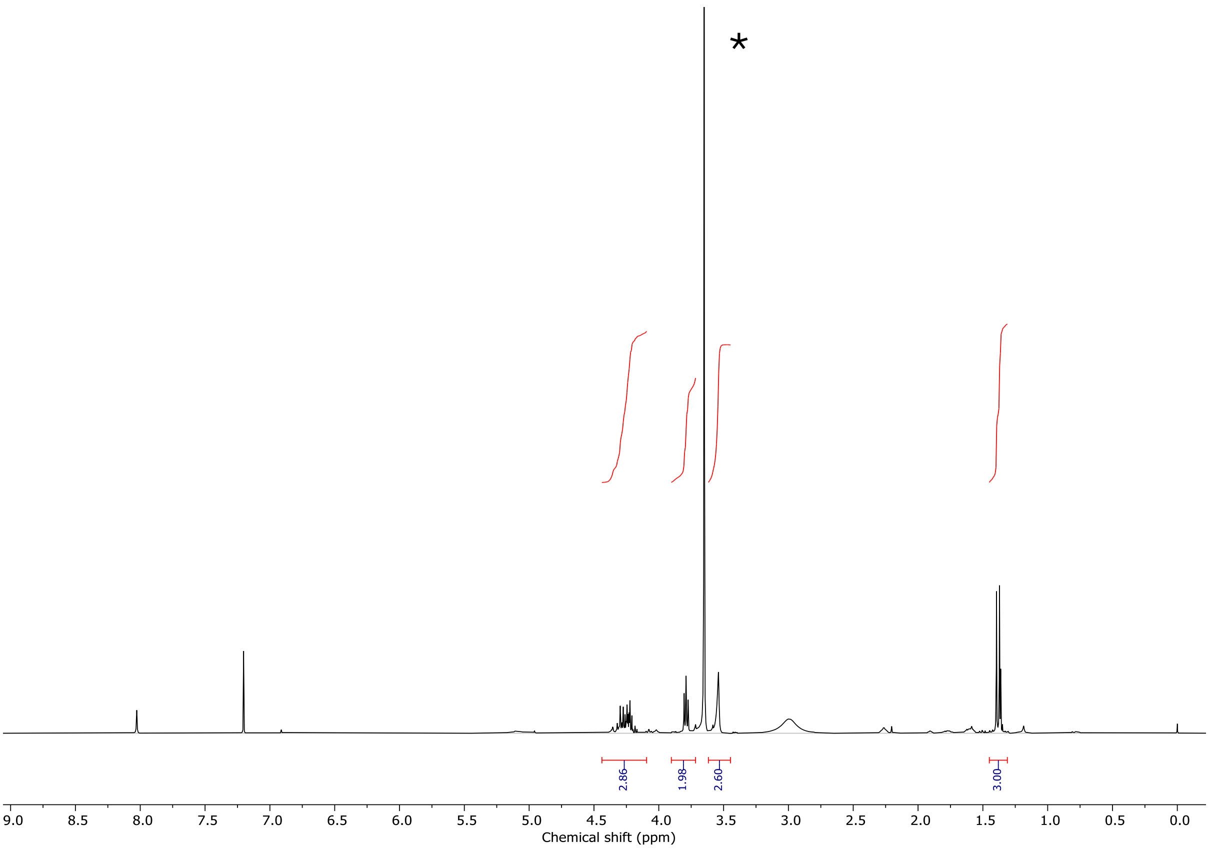

Figure S24. ^{1}H NMR ($CHCl_3$, 400 MHz) spectra of the reaction crude of the depolymerization of PLA straw waste under continuous flow. * = Ethylene Glycol.

References

[1] Petrus, R.; Bykowski, D.; Sobota, P. *ACS Catal.* **2016**, *6* (8), 5222−5235.

[2] Sardon, H.; Jehanno, C.; Demarteau, J.; Mantione, D.; Arno, C.; Ruiperez, F.; Hedrick, J.; Dove, A. *Angew. Chem. Int. Ed.* **2021**, *60* (12), 6710–6717.
